# Dissecting Tissue-Specific Transcriptomic Responses from Leaf and Roots under Salt Stress in *Petunia hybrida* Mitchell

**DOI:** 10.3390/genes8080195

**Published:** 2017-08-03

**Authors:** Gonzalo H. Villarino, Qiwen Hu, Michael J. Scanlon, Lukas Mueller, Aureliano Bombarely, Neil S. Mattson

**Affiliations:** 1 School of Integrative Plant Science, Cornell University, Ithaca, NY 14853, USA; gonzalo.villarino@yale.edu (G.H.V.); mjs298@cornell.edu (M.J.S.); 2 Institute for Translational Medicine and Therapeutics (ITMAT), University of Pennsylvania, Philadelphia, PA 19104, USA; qiwenhu@upenn.edu; 3 Boyce Thompson Institute, Cornell University, Ithaca, NY 14853, USA; lam87@cornell.edu; 4 Department of Horticulture, Virginia Polytechnic Institute and State University, Blacksburg, VA 24061, USA; aurebg@vt.edu

**Keywords:** salinity, salt stress, petunia, RNA-seq transcriptomic, trehalose, yeast complementation

## Abstract

One of the primary objectives of plant biotechnology is to increase resistance to abiotic stresses, such as salinity. Salinity is a major abiotic stress and increasing crop resistant to salt continues to the present day as a major challenge. Salt stress disturbs cellular environment leading to protein misfolding, affecting normal plant growth and causing agricultural losses worldwide. The advent of state-of-the-art technologies such as high throughput mRNA sequencing (RNA-seq) has revolutionized whole-transcriptome analysis by allowing, with high precision, to measure changes in gene expression. In this work, we used tissue-specific RNA-seq to gain insight into the *Petunia hybrida* transcriptional responses under NaCl stress using a controlled hydroponic system. Roots and leaves samples were taken from a continuum of 48 h of acute 150 mM NaCl. This analysis revealed a set of tissue and time point specific differentially expressed genes, such as genes related to transport, signal transduction, ion homeostasis as well as novel and undescribed genes, such as Peaxi162Scf00003g04130 and Peaxi162Scf00589g00323 expressed only in roots under salt stress. In this work, we identified early and late expressed genes in response to salt stress while providing a core of differentially express genes across all time points and tissues, including the trehalose-6-phosphate synthase 1 (*TPS1*), a glycosyltransferase reported in salt tolerance in other species. To test the function of the novel petunia *TPS1* allele, we cloned and showed that *TPS1* is a functional plant gene capable of complementing the trehalose biosynthesis pathway in a yeast *tps1* mutant. The list of candidate genes to enhance salt tolerance provided in this work constitutes a major effort to better understand the detrimental effects of salinity in petunia with direct implications for other economically important Solanaceous species.

## 1. Introduction

Salinity rapidly reduces plant growth inducing a suite of metabolic changes in plant physiology [1]. Initially upon sudden increases in sodium chloride, hormonal signals generated by the roots can lead to rapid reductions in growth and ultimately loss of yield in agriculture crops [2]. Salt-affected soils have become a major concern worldwide due to its detrimental impact in agricultural crop productivity. High rhizosphere NaCl levels can cause plant osmotic stress, protein misfolding, ion toxicity, nutritional deficiencies and oxidative stress among others [2]. The widespread effect of salinity accounts for 6% of the world’s total land area (over 800 million ha) [3]. Therefore, there is a necessity to improve the abiotic stress tolerance of agronomic and ornamental crops [4]. 

Studies of plant molecular responses to NaCl stress have focused mostly on model species such as *Arabidopsis thaliana*, providing valuable insights regarding mechanism for salt tolerance, such as salt exclusion by minimizing salt entry into the roots of plant, increased tolerance by the expression of antioxidant enzymes, heat shock proteins and compartmentalization of Na^+^ ions in the vacuole of cells [5]. However, *A. thaliana*, a glycophyte species, is sensitive to moderate levels of NaCl and therefore it is difficult to explore novel processes or mechanisms naturally occurring in stress-tolerant plants [2,6].

*Petunia hybrida* belongs to the Solanaceae family, a highly diversified group with more than 3000 species including major crops such as *Solanum lycopersicum* (tomato), *Solanum tuberosum* (potato), *Capsicum annuum* (pepper) and *Nicotiana benthamiana* (tobacco), representing a diverse and economically important group of agriculture crops worldwide [7]. In the U.S. alone annual wholesale value of tomato, potato, pepper, tobacco, and petunia is 2.3, 4.0, 0.8, 1.3 and 0.13 billion respectively [8,9]. Solanaceous plants provide important model systems for both genetic and biochemical studies such as tomato and pepper (fruit development), potato (tuber development), tomato and tobacco (plant defense against herbivores) and petunia (flower development and senescence) [10].

Petunia is an emerging new model for salt stress as it is a species that can withstand short-term high-level salt stress (80 mM NaCl) without lethal consequences, exhibiting only smaller plant size and some chlorosis in leaf edges, but maintaining growth and development [11]. 

Salt tolerance is the result of complex genetic interactions controlled by quantitative trait loci [12] where the plant response to salt will usually involve changes in the expression of hundreds, if not thousands, of genes [13,14]. Despite the importance of Solanaceae as crops and model plants, there have not been many comprehensive and/or integrated studies with these species under salt stress. 

Efforts to study the broad effects of NaCl in plants have been carried out in different species using transcriptomic [15,16] and genomic approaches by next generation sequencing (NGS) techniques, RNA sequencing (RNA-seq) in particular [17]. 

Different NGS platforms (i.e., Illumina and 454 sequencing) have been used to study salt stress due to the improved sensitivity, wider dynamic range and better accuracy for quantifying expression levels with RNA-seq versus previous methodology for RNA profiling such as microarray, Northern blots, expressed sequence tags (ESTs) and serial analysis of gene expression (SAGE) [18]. Vinocur et al., reported a range of metabolites, including amino acids (e.g., proline) and sugars and sugar alcohols (e.g., trehalose), that can prevent these detrimental consequences of NaCl [19].

To complement previous research carried out with *P. hybrida* under salt stress [15], paired-end RNA sequencing libraries spanning 24 h of acute salt stress from leaves and 48 h of acute salt stress from roots were sequenced and analyzed. Over a thousand genes were differentially expressed through the course of 24 h and 48 h in both leaves and roots tissues. Some of the most differentially expressed genes were phosphatases, expansin-like proteins, non-specific lipid transfer proteins, MYB transcription factors and synthesis of sugars such as *galactinol synthase 1* and *glycerol-3-*phosphate acyltransferase and the trehalose-6-phosphate synthase (*TPS1*). Of particular interest is the *TPS1* gene, which was up-regulated in all time points and all tissues and has involved in coping with salt stress in Arabidopsis [20,21], as well as the phosphatidylinositol-4-phosphate 5-kinase (*PIP5K*) gene, required in signal transduction pathways, induced over 60-fold under 24 h of NaCl stress. 

In this study, a suite of candidate genes is provided aiming to potentially enhance salt tolerance by genetic engineering approaches. To the best of our knowledge, this work provides the most comprehensive transcriptomic analysis of any Solanaceous species to salt stress.

## 2. Materials and Methods

### 2.1. Plant Material and Treatments

Sixty seedlings of *P. hybrida* cv. ‘Mitchell Diploid’ (a doubled haploid derived from *Petunia axillaris* and *P. hybrida* cv. ‘Rose of Heaven’) [22] were germinated for 3 weeks in a soilless substrate. Of those, 20 seedlings were selected for uniformity, i.e., similar size (ca. 8 cm height), number of branches, absence of biotic or abiotic disorders and same development stage—first flower initiation) and transferred to 4 L containers in solution culture and placed in a growth chamber at 22 °C and 200 µmol m^−2^ s^−1^ Photosynthetically active radiation (PAR) for 12 h daily. Containers were randomly distributed. In each container, a modified Hoagland’s nutrient solution (4 mM KNO_3_, 1 mM MgSO_4_, 1 mM NH_4_H_2_PO_4_, 4 mM Ca(NO_3_)_2_∙4H_2_O, 18 µM Fe-EDDHA, 2 µM CuSO_4_·5H_2_O, 4 µM ZnSO_4_·7H_2_O, 0.2 µM H_2_MoO_4_·H_2_O, 28 µM MnCl_2_·4H_2_O, 4 µM H_3_BO_3_) prepared in reverse osmosis filtered water was kept aerated using an aquarium pump. After acclimation to the growth chamber (7 days) the most representative 18 plants were divided into two treatment groups with nine containers each and again randomly distributed throughout the growth chamber. The control (referred to as “CTR”) group received the Hoagland’s solution with no added NaCl and the salt treatment (referred to as “STR”) group received Hoagland’s solution amended with 150 mM NaCl. 

### 2.2. Tissue Sample and RNA Isolation

From within the nine plants per treatment, three biological replicates were established by randomly grouping three sets of plants within each treatment condition. At each time point (as described below) samples from three plants per group were pooled together to create one biological replicate. Leaves were sampled at 0, 6, and 24 h and roots were sampled at 0, 6, 12, 24 and 48 h after treatment was applied (NaCl vs. Hoagland’s solution with no added NaCl), yielding 48 samples total (8 time points and organs × 2 treatments × 3 biological replicates). At each time point roots were carefully dissected longitudinally (i.e., strands of full length roots from the base of the plant to the root tip). The most recently expanded leaf (fourth or fifth leaf from the lateral meristem) from a lateral branch was selected. It is important to note that plants did not have enough of the targeted mature leaves to take from more than three time points (while they had plenty of root samples to take more time points), which left us with more roots than leaves time points, in addition this is why plants were grouped into three sets within each treatment, with one plant within each set sampled at each time point. The 48 samples were frozen immediately in liquid nitrogen and stored at −80 °C before RNA isolation. Total RNA was isolated using Trizol Reagent (Invitrogen, Carlsbad, CA, USA) and purified through a Qiagen RNeasy Column (Qiagen, Hilden, Germany) according to the manufacturer’s instructions. A 1% agarose gel was run to indicate the integrity of the RNA and ribosomal bands were used for total RNA quality control. Four root and leaf samples were further quantified in an Agilent 2100 Bioanalyzer (Agilent, Santa Clara, CA, USA) at the Cornell University Biotechnology Resource Center) to verify total RNA quality. RNA integrity number (RIN) for these samples analyzed were 8.5, 9.1, 8.9, 8.5 (roots), 8.7, 8.5, 8.7 and 6.7 (leaves).

### 2.3. Library Preparation and Deep Sequencing

Multiplexed libraries for the 48 samples were constructed using a High-Throughput Illumina Strand-Specific RNA Sequencing Library protocol [23]. The forty-eight double stranded complementary DNA (cDNA) libraries were pooled together (20 ng/library) and sent for sequencing to the Cornell University Biotechnology Resource Center (http://www.biotech.cornell.edu/biotechnology-resource-center-brc).

Paired-end sequencing was performed including 2 × 100 cycles + 7 cycle index-read in in 3 lanes of the HiSeq 2500 Illumina platform with ‘TruSeq PE Cluster Kit v3’ for the flow-cell and ‘TruSeq SBS kit v3’ for the sequencing reagents. 

### 2.4. Processing of Illumina RNA-Seq Reads

Quality control and preprocessing of metagenomic data were performed using FastQC software [24]. Adapters and low-quality sequences were filtered out with Ea-Utils software [25]. Reads with phred-like quality score greater than 30 and read length greater than 50 bp were kept and aligned against the *P. axillaris* reference genome [26]. The *P. axillaris* genome was used as a reference as *P. hybrida* is more closely related to *P. axillaris* than its other parental species *Petunia inflata*. 

### 2.5. Sequence Alignment to the Petunia Genome

The splice junction mapper TopHat2 [27] was used to align filtered RNA-seq reads to the *Petunia axillaris* genome. Default parameters for TopHat2 were used except for strand specificity (–library-type = fr-firststrand) to match to the first strand of cDNA synthesized (antisense to the mRNA). Uniquely mapped reads were extracted from the TopHat2 output binary (BAM) file using samtools [28] and selected for the “NH:i:1” two-character string tag. Only uniquely mapped reads were used for downstream analysis.

### 2.6. Table Counts

The HTSeq: Analyzing High-Throughput Sequencing Data with Pythons software [29] was used with default parameters except for the stranded = reverse and “-i ID” mode to generate tables-counts for downstream differential expression analysis for the R packages edgeR [30]. 

### 2.7. Gene Expression and Differential Gene Expression

We performed gene expression and differential gene expression analyses with the R packages edgeR [30] and the Linux-based Cufflinks program [31] (version 2.2.1) “-G” option. With edgeR, we discarded genes whose cpm (counts per million) was lower than a threshold of two reads per gene in at least three biological replicates, as suggested in the edgeR vignette [32]. To identify expressed gene with Cufflinks, we chose those genes that had a lower confidence interval bigger than 0 (conf_lo > 0) and whose status was “ok” (quant_status = “OK”).

### 2.8. Bar Plots, Venn Diagrams and Heat Maps

Graphs were built using the R packages “bear” (https://cran.r-project.org/src/contrib/Archive/bear/) and “plyr” (https://cran.r-project.org/web/packages/plyr/index.html) to calculate means, SE, and confidence intervals and ggplot2 (https://cran.r-project.org/web/packages/ggplot2/index.html) to generate the plots. Venn diagrams were constructed using the R package “VennDiagram” [33]. Heat maps were produced using the R package “pheatmap” (https://cran.r-project.org/web/packages/pheatmap/index.html) FPKM normalization by gene length and library size (Cufflinks) was used. Samples were clustered (default clustering) with parameters provided in the software. The R package colorRamp (https://cran.r-project.org/web/packages/colorRamps/index.html) was used to produce a gradient of color values corresponding to gene fold change values.

### 2.9. Sequencing Data

Illumina sequencing raw data (fastq) and table counts have been submitted to the Gene Expression Omnibus (GEO) database (accession no. GSE97428).

### 2.10. Phylogenetic Analysis

Alignments of amino acid sequences *Nicotiana tabacum*, *S. lycopersicum*, *Vitis vinifera*, *Populus trichocarpa*, *A. thaliana*, *Ricinus communis*, *Sorghum bicolor*, *Zea mays*, *Oryza sativa*, *Triticum aestivum*, *Selaginella lepidophylla*, *Candida albicans*, *Saccharomyces cerevisiae*, *Clavispora lusitaniae*, *Apis mellifera*, *Drosophila melanogaster*, *Escherichia coli* and *Ginkgo biloba* was performed via pairwise and multiple sequence alignment algorithms (MUSCLE) using DNASTAR’s Lasergene Molecular Biology Suite software. 

### 2.11. Truncating the Petunia Trehalose 6 Phosphate Synthase PhTPS1 (*⊗*PhTPS1)

One of the highly differentially expressed genes in our dataset was the *TPS1*, which has also been implicated in salt stress tolerance in other species [20,21]. To verify function of the *TPS1* we cloned and tested its function in a yeast system. Analysis of the *TPS1* N-terminus from *A. thaliana* and *S. lepidophylla* led to the identification of two conserved residues which decrease enzyme activity when present [20]. Therefore, we truncated the first ~80 amino acids (aa) towards the 5′-end to increase the *PhTPS1* catalytic activity. Two different primers were designed to truncate the 3 kb *PhTPS1* while at the same time introducing sequence homologous to the promoter of the pDB20 vector, used in later cloning steps. A 56 bp forward primer (5′-*GCTATACCAAGCATACAATCAAC*TTAAAGCGGCCGC**ATG**CAACGACTCTTAGTTGT-3’) was designed. The 5’-end of this primer has 23 bp of the promoter sequence of pDB20 vector (*italics*) followed by five bases of filler sequence (TTAAA), a NotI restriction site (underlined), an in-framed ATG start codon (bold case) and 17 bases complementary to the *PhTPS1* sequence. A reverse primer (5′- *TGGCGAAGAAGTCCAAAGCTT*ATTTGCGGCCGC*TTA*AGAAGAGGCTTCAGCTAGT-3’) including a stop codon was used. This trimmed petunia *TPS1* gene (⊗*PhTPS1*), which now included a yeast promoter sequence, was cloned into the pDB20 yeast vector and sequenced. 

### 2.12. The pDB20 Yeast Vector

The backbone of the pDB20 yeast expression vector comes from the pUC18 vector. pDB20 carries the ADCI (ADHJ) promoter which drives high levels of gene expression. In the polylinker region this vector has six cloning sites; HindIII/NotI/BstXI/BstXI/NotI/HindIII. The vector has URA3 as a yeast selectable marker and amp^R^ as an *E. coli* selectable marker. The vector also has HpaI restriction site outside the polylinker region.

### 2.13. Cloning *⊗*PhTPS1 into the pDB20 Yeast Expression Vector

0.25 µg of pDB20 digested with NotI (Invitrogen was treated with phosphatase following the protocol provided by the New England BioLabs kit (Antarctic Phosphatase, New England BioLabs, Ipswich, MA, USA). The trimmed ⊗*PhTPS1* (0.23 µg), was digested with the same NotI enzyme. Ligation was carried out with T4 DNA ligase enzyme (Lucigen, Middleton, WI, USA) and the ligation mix was used to transform competent *E. coli* cells (Lucigen, *E. cloni* 10G CLASSIC electrocompetent cells). Plasmid was fully sequenced to check for correct open reading frame (ORF) and rule out PCR artifacts. 

### 2.14. Yeast Strains and Functional *TPS1* Gene Analysis

Four *S. cerevisiae* strains were used for the yeast complementation assay. They were received from the ‘VIB laboratory of Molecular Cell Biology (K.U. Leuven)’ Flanders, Belgium. Yeast genotypes are as follow: wild type ‘W303-1A’ (Mat a leu2-3, 112ura3-1, trp1-1, his3-11, 15 ade2-1, can1-100, GAL, SUC2), *tps1*⊗ ‘YSH290’ (W303-1A, ggs1/*tps1*⊗), *tps2*⊗ ‘YSH450’ (W303-1A, *tps2*⊗:LEU2,RP1) and a double knockout *tps1*⊗*tps2*⊗ ‘YSH652’ (W303-1A, ggs1/*tps1*⊗:TRP1, *tps2*⊗:LEU2). 

Yeast strains were grown in complete liquid medium containing glucose (YPD: 10 g peptone, 20 g yeast extract, 20 g dextrose and 1L dH_2_O). Cells were transformed **(**Frozen-EZ Yeast Transformation II Kit, Zymo research, Irvine, CA, USA) with 0.25 µg of the NotI-digested pDB20 vector plus 0.23 µg of ⊗*PhTPS1* DNA. Another aliquot of the competent cells was transformed separately with NotI-digested pDB20 vector. The transformed yeast was plated on complete supplement mixture–Ura (CSM–Ura; Yeast nitrogen base without amino acids, glucose, agar and Complete Supplement Mixture minus uracil; Sunrise Science Products, San Diego, CA, USA) media to select for Ura^+^ cells. Cells from W303-1A (WT), YSH290 (*tps1*⊗), YSH450 (*tps2*⊗) and YSH652 (*tps1*⊗*tps2*⊗) with pDB20^+^⊗*PhTPS1* and empty pDB20 vector alone were streaked onto Min + Leu + His + Ade-Ura + Galactose and onto Min + Leu + His + Ade-Ura + Glucose to assess if the ⊗*PhTPS1* would restore the function of mutant yeast while growing on glucose. 

## 3. Results and Discussion

To investigate the transcriptomic profile of petunia under salt stress, we performed high-throughput RNA-seq from leaves and roots across 150 mM NaCl. We expected that the identification of differentially expressed genes (DEGs) between the tissues and time points will provide insight into the set of transcripts expressed at early and late stages of salt stress, as well as those DEGs expressed in all samples.

Three lanes of the HiSeq2500 Illumina sequencing platform yielded ~700 million paired-end raw reads, with an average of ~14 million reads per library. Nearly 20 million reads were filtered out after removing barcode adapters and low-quality sequences. The remaining 672 million reads were aligned against the newly sequenced *P. axillaris* reference genome [26] with more than 75% of them successfully mapping to the genome sequence. Among the ~600 million reads mapped to the genome, 532 million were mapped uniquely to only one location and used for subsequent downstream analyses. A detailed breakdown is shown in Table S1.

We used two independent and open-source programs for RNA-seq analysis to determine expressed and DEGs in our data set: Cufflinks [31] and edgeR [30]. Our choice of these tools reflects significant differences in the underlying algorithms; while Cufflinks uses isoform expression estimates, edgeR compute gene level-based expression estimates. Furthermore, as they use different normalization approaches and dispersion estimates, we expected each program to return a somewhat different set of DEGs. Indeed, edgeR was generally more conservative in identifying DEGs at the same cutoff than Cufflinks (Figure S1).

### 3.1. Transcriptomic Relationship between the Samples

To assess the transcriptomic relationship between the samples, we used hierarchy clustering with gene-expression values. Hierarchical clustering by Pearson correlation distance method shows that samples cluster, first, by tissue (leaf vs. roots) and then based on responses to salt stress rather than time point (Figure S2A). We also employed a Spearman rank correlation (as it is less sensitive than the Pearson correlation to strong outliers) and does not inflate type I error rates [34,35]. This approach is in accordance with Pearson; samples cluster based on tissue and responses to salt stress (Figure S2B). To facilitate future use of these datasets, all the expressed genes identified by both program in all time points and tissue samples are included (Tables S2–S9).

### 3.2. Differentially Expressed Genes across Time Point and Tissues

**Figure 1:**
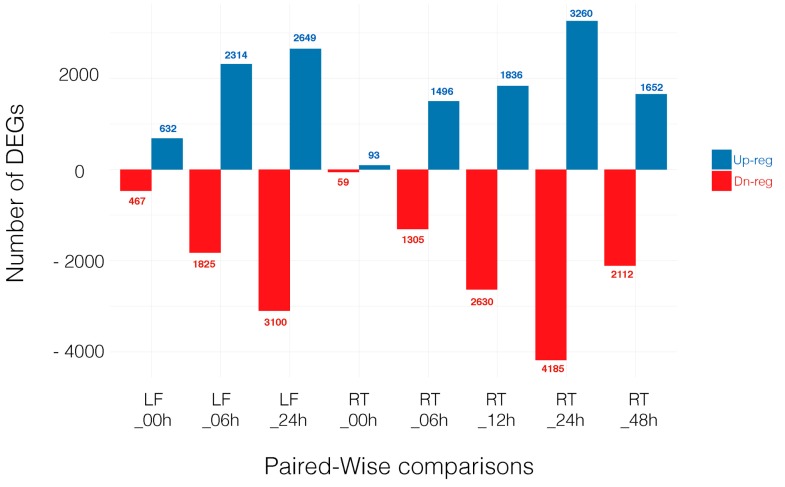
Number of differentially expressed genes (DEGs) that are upregulated (Up-reg) and downregulated (Dn-reg) in each time point comparison. LF: Leaf; RT: Root.

For a gene to be selected as DEGs, we required the transcript to be identified by both edgeR and Cufflinks at a false discovery rate (FDR) < 0.05. The number of DEGs is shown in the overlap sections in the Venn diagram, Figure S1. Note that hereafter we focused on those eight comparisons where genes from a given tissue at a specific time point in the control treatment (CTR) is compared with the same tissue at the same time point under salt stress (STR). Thus, differential gene expression is based on treatment (control vs. salt) and not other variables (see Materials and Methods section). Throughout the manuscript we refer to these comparisons as: LF_00h, LF_06h, LF_24h, RT_00h, RT_06h, RT_12h, RT_24h and RT_48h. The number of up- and downregulated genes in each comparison is shown in Figure 1 and all DEGs can be found in Tables S10–S17. 

### 3.3. Expanding from Previous Transcriptomic Studies

We previously performed a similar experiment where petunia plants were exposed to NaCl stress and leaf samples were sequenced [15]. Although an RNA-seq de novo assembly approach was used to map reads instead of the petunia reference genome, some of the DEGs reported here were also identified in our previous study. In both approaches, genes involved in sugar synthesis were highly expressed in leaves. For example, the bidirectional sugar transporter (*Peaxi162Scf01337g00018*), and α-glucan water dikinase (*Peaxi162Scf00192g00217*) were identified in leaves with a fold induction of >4 (24 h) and 8-fold induction in leaf at 6 h of salt stress. Moreover, sugar synthesis-related genes were identified, such as galactinol synthase (*Peaxi162Scf00366g00813*) as well as chaperone genes from the 60 and 70 kDa family were differentially expressed in leaf tissue and detected with both approaches. 

To gain insight into salt stress in a tissue specific manner, in addition to leaves, we added root samples and used the petunia genome to map reads. Interestingly, a highly expressed root specific gene was identified in this work. The phosphatidylinositol-4-phosphate 5-kinase (PIP5K) (*Peaxi162Scf00258g00045*) was significantly induced in all root time points; 12-fold at 6 and 12 h, 60-fold at 24 h, and 18-fold at 48 h. Notably, chemically related to *PIP5K*, the inositol polyphosphate 5-phosphatase I (*Peaxi162Scf00422g00510*) was highly expressed (>10-fold, FDR > 0.01) only in roots at 48 h, suggesting that the phosphatidylinositols family of lipids are an essential class of lipids with important roles in early and late salt stress [36]. Although not well characterized in plant cells, phosphoinositide signaling pathways have been linked to abiotic stress such as salinity and drought [37].

PIP5K phosphorylate phosphatidyl inositols (PtdIns) into phosphatidyl inositol bisphosphates PtdIns(4,5)P2, an important substrate for hydrolysis generating 1,2-diacyglycerol (DAG) and inositol 1,4,5-trisphosphate (IP3) [38]. IP3 acts as a secondary messenger in the transduction of stress signals opening calcium channels on the smooth endoplasmic reticulum (SER), allowing calcium ion mobilization through specific Ca^2+^ channels into the cytosol [39]. Rapid increase in cytosolic calcium under salt stress has been reported in several studies [40,41]. 

These results for *PIPK5* are in accordance with those of DeWald et al. [36], who demonstrated that plants respond to salt and osmotic stress by synthesizing phosphoinositides. In their work, 2-week-old *A. thaliana* plants were treated (immersed) in osmotic-adjusting solutions with 250 mM NaCl for 1 h. High performance liquid chromatography (HPLC) analysis revealed that glycerophosphoinositol phosphate compounds increased by approximately 20-fold in immersed plants vs. non-stressed plants. 

Apse et al. showed that overexpressing a vacuolar antiport in *Arabidopsis* increased the ability to withstand high levels of salt (up to 200 mM NaCl) [42]. In our dataset, the cation/H^+^antiporter 15 (*Peaxi162Scf00284g00220*), was highly induced in roots at 24 h (>12-fold) and at RT_48h (>7-fold). We also identified the *Peaxi162Scf00192g00922* (K^+^ efflux antiporter 5) that significantly increased its expression in RT_48h (>3-fold). 

Master regulators such as MYB transcription factors also play a key role in salinity tolerance, as suggested in this work. *Peaxi162Scf00147g00136* (myb domain protein 12) was significantly induced (FDR < 0.01) (Tables S2–S17). Different MYB members mediate signal transduction and regulate some stress-responsive genes involved in NaCl stress coping mechanism [43,44].

**Figure 2:**
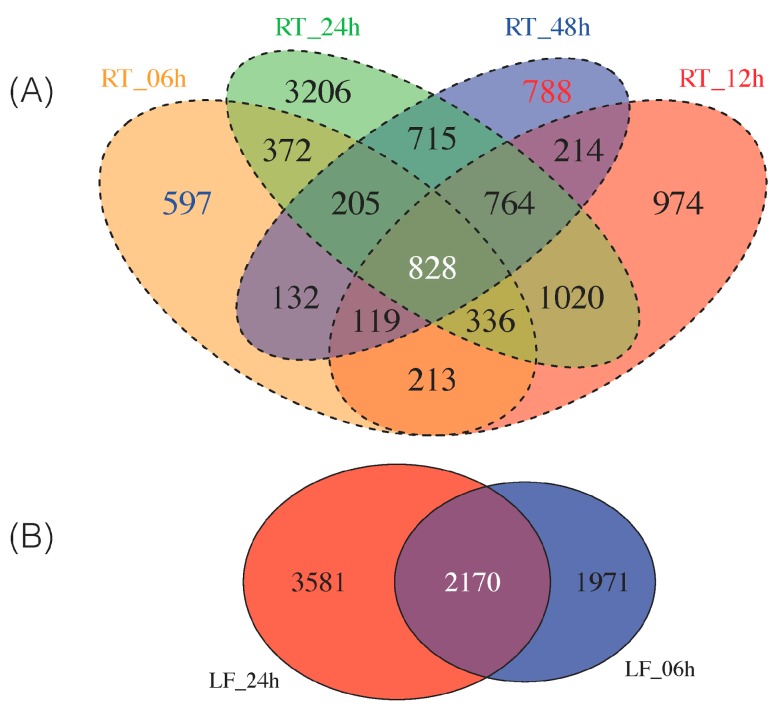
Commonly identified DEGs by Cufflinks and edgeR in all samples at all time points (FDR (false discovery rate) < 0.05). (**A**) Four-way Venn diagram of all the DEGs identified from roots at time points 0, 6, 12, 24, 48 h. Early (6 h) expressed genes are highlighted in blue and late (48 h) expressed genes are highlighted in red; (**B**) Two-way Venn diagram of all DEGs from leaves at time points 0, 6 and 24 h.

As some of these DEGs are exclusively expressed at early (i.e., 6 h) and late (i.e., 48 h) time points in the 48 h continuum of NaCl stress in roots, we sought to classify which DEGs were up- and downregulated as early/late response. Thus, we identified 597 DEGs solely expressed at 6 h and 788 DEGs as late (48 h) response (Figure 2A). We provide the top 10 most highly up-/downregulated DEGs from roots at 6 h and 48 h in Table 1. All these DEGs can be found in Tables S18–S19.

### 3.4. Candidate Genes to Enhance Salt Stress

To this end, we sought to identify specific candidate genes that can potentially increase salt stress tolerance in plants. We reasoned that good candidate genes were those whose steady-state transcript levels are significant at the early onset of salt stress (i.e., 6 h) and whose expression remained active through days of exposure to NaCl stress (i.e., 48 h) in both leaves and roots. 

Thus, a stringent criterion was used to select a subset of leaf and roots DEGs for downstream analysis; we required the transcript to be identified as differentially expressed in all six comparisons (LF_06h, LF_24h, RT_06h, RT_12h, RT_24h, RT_48h) by both edgeR and Cufflinks. Using this approach, as suggested by others [45], led to the identification of 828 root DEGs and 2170 leaf DEGs (Figure 2). We then overlapped the 828 and 2170 DEGs to generate a final common list of 178 genes differentially expressed in both roots and leaves over a 48 h period (Figure S3B).

As no salt stress is induced at 0 h, we sought to confirm that this common list of 178 genes were not differentially expressed as a result of stresses other than NaCl, such as mechanical damage and/or changes in growing conditions (adapting to hydroponic system). As a conservative estimate of the control gene set, we identified transcripts in the union of all the DEGs at 0 h in both roots and leaves. Thus, we would consider a “control” gene even if it was differentially expressed in only one tissue (Figure 4a). This lead to 1291 “control” DEGs expressed at 0 h (Table S20).

We expected a small overlap between the set of control DEGs identified either in roots and/or leaves at 0 h versus those DEGs identified from tissues exposed to NaCl stress. Indeed, only 18 transcripts were found in common when overlapping these datasets (Figure S3C.). We then removed these 18 transcripts from our analysis to eliminate any gene that might be differentially expressed as a result of other stresses than NaCl, yielding a final “cleaned” list of 160 DEGs (Table S21).

**Figure 3:**
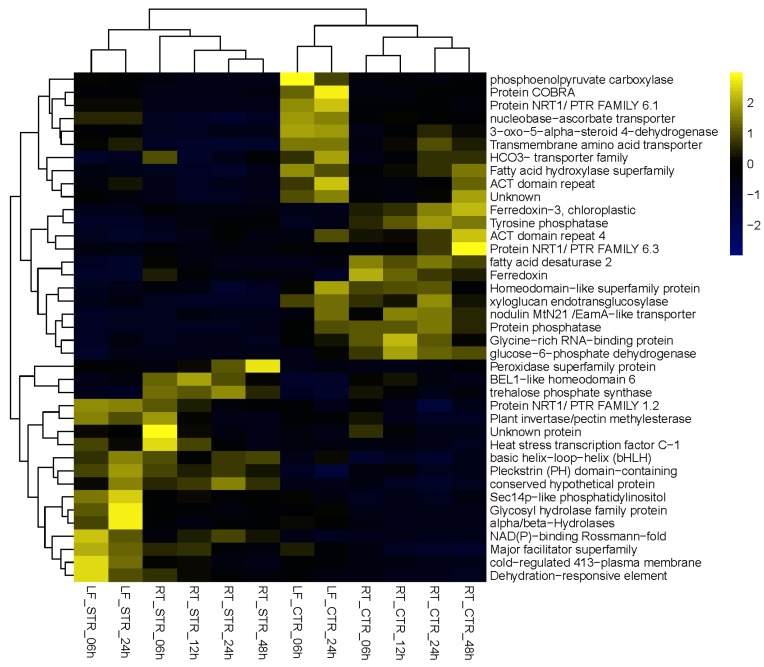
Heatmap representation of the expression profiles of the 17 up- and 22 downregulated DEGs from the 160 DEGs.

Although many of these 160 DEGs were up/down regulated based on time points and tissue, we found 17 DEGs in the dataset of 160 genes that are induced at all time points (6, 12, 24, and 48 h in roots; and 6 and 24 h in leaves) and 20 DEGs whose transcript levels were downregulated at all time points in all tissues. The expression profile of these up (17) and down (20) regulated DEGs is represented in a heatmap (Figure 3) and the top 10 most up- and downregulated from the 160 DEGs are shown in Table 2.

### 3.5. Petunia Trehalose-6-Phosphate Synthase 1 Gene, a Candidate Gene to Enhance Salt Tolerance in Solanaceae Plants

**Figure 4:**
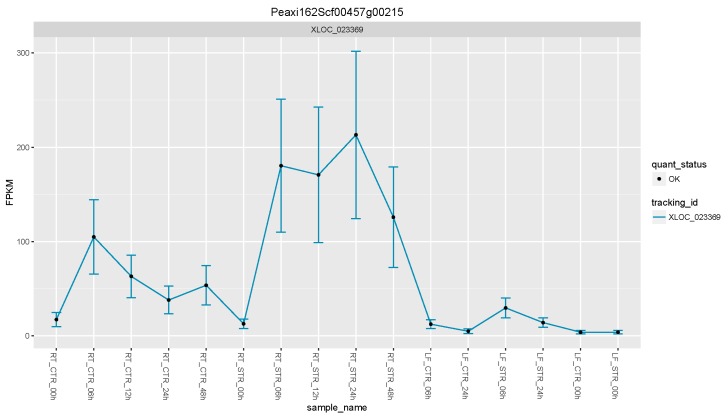
Expression levels of the *PhTPS1* gene across all time point and tissues. FPKM: Fragments per kilobase of exon per million fragments mapped.

Using this approach, we were able to select a suite of potential salt gene regulators. From the list of the 17 upregulated DEGs (Figure 3 and Table 2), the petunia trehalose-6-phosphate synthase (*PhTPS1*) gene increased its expression across all the time points, with maximum expression values in roots at 24 h of salt stress (Figure 4).

Trehalose sugar, widely present in bacteria, yeast and some plants, prevents physical and chemical instability in proteins that occurs upon desiccation when exposed to high concentration of salt [46]. In the first step of the trehalose biosynthesis pathway, the trehalose-6-phosphate synthase gene (*TPS*) form α,α-trehalose 6-phosphate (T6P) intermediate sugar which is then converted to trehalose by a trehalose-phosphatase gene (*TPP*) [47]. It has also been hypothesized the T6P as a sugar signaling molecule whose synthesis and degradation is regulated for coordinated pathways in response to prevailing carbon supply [48].

Inactivation of the *S. cerevisiae* TPS gene (*ScTPS1*) causes a growth defect when grown in the presence of glucose in the medium associated with deregulation of the initial part of glycolysis. Because glycolysis is essential in all eukaryote organisms [49], we next verified if the *PhTPS1* was a functional gene capable of complementing the trehalose biosynthesis in yeast.

### 3.6. The Petunia Trehalose-6-Phosphate Synthase 1 Gene Rescue Mutant Yeast Phenotype

We cloned the novel *PhTPS1* allele and performed a phylogenic analysis based on amino acid sequences. The predicted amino acid sequence from the petunia cDNA confirms that the *PhTPS1* protein is most closely related to the Solanaceae family, as shown in the phylogenetic tree (Figure S4).

We next performed a functionality assay with the *PhTPS1* to assess if the plant allele could directly rescue yeast mutant phenotypes. We truncated the first 80 aa towards the 5′-end of the *PhTPS1* to increase its catalytic activity, as it has been previously shown for bacterial genes (see Materials and Methods section). We used four different yeast strains; two single knockout mutants (“*tps1*⊗” and “*tpp*⊗”), a double knockout “*tps1*⊗*tpp*⊗” and a wild type yeast strain (see genotypes in Material and Methods section). We reasoned that the *TPS1* gene would rescue the lethal phenotype from the *tps1*⊗.

**Figure 5:**
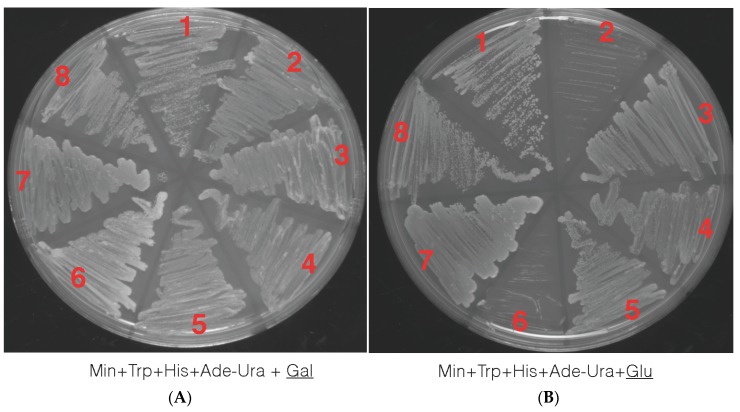
Yeast complementation studies with a carbon source of galactose (Gal, 5.A) and glucose (Glu, 5.B.) In both **A** and **B**, four different strains were transformed with empty vector (EV) or vector plus *PhTPS1* gene (V + G) and streaked onto Yeast Extract Peptone Dextrose (YPD) media to assess the ⊗*PhTPS*1 functionality. 1. YSH290 (*tps1*⊗) V + G. 2. YSH290 (*tps1*⊗) EV. 3. YSH450 (*tps2*⊗) V + G. 4 YSH450 (*tps2*⊗) EV. 5. YSH652 double knockout (*tps1*⊗*tps2*⊗) V + G. 6. YSH652 double knockout (*tps1*⊗*tps2*⊗) EV. 7. Wild type W303-1A V + G. 8. Wild type W303-1A EV. Only YSH290 (*tps1*⊗) V + G and YSH652 double knockout (*tps1*⊗*tps2*⊗) V + G were able to grow in glucose when looking at the *TPS1* allele showing that *PhTPS1* it is capable of restoring *TPS1* function in a mutant yeast. Empty vector alone failed to rescue function and YSH290 (*tps1*⊗) EV and YSH652 double knockout (*tps1*⊗*tps2*⊗) EV cells were not able to grow in glucose.

Yeast transformations were performed with the truncated petunia *TPS1* allele in the pDB20 yeast expression vector and the vector alone (as control) via yeast homologous recombination. Transformants were selected by plating on Min^+^ their respective amino acids minus Ura^+^ media to select for those that became prototrophic (^+^) for the ability to synthesize Ura^+^ marker (Material and Methods section). All four strains transformed with empty vector and the *PhTPS1* gene grew on galactose sugar when provided as carbon source, as expected (Figure 5A). However, when the carbon source was changed to glucose, neither the *tps1*⊗ single mutant nor the *tps1*⊗*tps2*⊗ that were transformed with the empty vector were able to grow. Conversely, the *tps1*⊗ and *tps1*⊗*tps2*⊗ complemented with the petunia *TPS1* gene was able to grow in glucose, showing that *PhTPS1* is a functional gene capable of restoring the influx of glucose into glycolysis. Empty vector and vector plus gene did not have a growth effect in the wild type W303-1A and *tps2*⊗ yeast background, as shown in Figure 5B.

Interestingly, the rate-limiting step in the trehalose biosynthesis appears to be the *TPS1* gene, as the double knockout vector plus *TPS* gene was able to grow in glucose. This in turn, suggests that the T6P intermediate can be dephosphorylated by nonspecific phosphatases. We believe that future research should aim to preform analysis in transgenic plants overexpressing the *PhTPS1,* as plants will most likely enhance their tolerance salt stress. In summary, this analysis reveals a suite of thousands of genes that are differentially expressed in *P. hybrida* in roots and leaves upon perceiving and responding to salt stress. For example, calcium-dependent protein kinases expression increased significantly upon acute salt stress, indicating that calcium plays an important role in early steps of the transduction pathway of salt stress signaling. Expression of genes such as the root specific *PIP5K* appear to provide a quick way to relay stress signals leading to downstream gene expression to mitigate salt damage. Master regulators such as MYB transcription factors also play a key role in salinity tolerance, as suggested in this work. Different MYB members mediate signal transduction and regulate some stress-responsive genes involved in NaCl stress coping mechanism. Importantly, the *TPS1* gene, widely described in the literature for its involvement in abiotic stress tolerance in other species, was differentially expressed at all time points in all tissue and, upon functional assay we showed is a functional gene capable of rescuing mutant yeast phenotype. Although we focused on a subset of genes, it is important to note that other DEGs identified in this work should not be discounted as potential salt stress regulators. Other approaches in the near future may lead to the discovery of other putative enhancer of salt stress.

## Figures and Tables

**Table 1:** Early (6 h) and late (48 h) significantly (FDR < 0.05) expressed genes in petunia roots identified by edgeR and Cufflinks.

	**Gene Short Name**	**Annotation**	**Cufflinks**					**edgeR**				**Arabidopsis Homolog**	**^3^IPR**	**^4^GO**
			**RT_CTR_06h**	**RT_STR_06h**	**Fold Change**	***p*-Value**	***p*-Value**	**Fold Change**	**^1^LR**	***p*-Value**	**^2^FDR**			
6h-Up Reg	*Peaxi162Scf00500g00067*	Late embryogenesis abundant protein, putative	0.23	10.82	46.54	2.60E-03	6.61E-03	84.23	62.39	2.82E-15	2.11E-13	AT3G53040.1	IPR004238	
6h-Up Reg	*Peaxi162Scf00845g00031*	Zinc finger CCCH domain-containing protein 2	0.36	8.43	23.11	2.20E-02	4.21E-02	122.75	114.94	8.09E-27	3.64E-24	sp|Q9ZWA1|C3H2_ARATH	IPR000571	GO:0046872
6h-Up Reg	*Peaxi162Scf00921g00256*	Protein NRT1/PTR FAMILY 6.4	0.29	6.13	20.91	7.90E-03	1.74E-02	107.55	43.79	3.66E-11	1.25E-09	sp|Q9LVE0|PTR33_ARATH	IPR000109,IPR016196	GO:0016020,GO:0006810,GO:0005215
6h-Up Reg	*Peaxi162Scf00366g00816*	Mannan endo-1,4-β-mannosidase 7	1.52	14.22	9.38	4.50E-04	1.39E-03	4.17	20.00	7.73E-06	7.88E-05	sp|Q9FJZ3|MAN7_ARATH	IPR017853	GO:0004553,GO:0005975,GO:0003824
6h-Up Reg	*Peaxi162Scf00166g00824*	Cyclic nucleotide-gated channel 14	0.40	3.68	9.31	4.00E-03	9.62E-03	5.88	22.42	2.19E-06	2.61E-05	AT2G24610.1	IPR014710	
6h-Up Reg	*Peaxi162Scf00002g00037*	Myb domain protein 42	0.73	6.32	8.65	2.24E-02	4.28E-02	6.34	21.16	4.23E-06	4.65E-05	AT4G12350.1	IPR009057	GO:0003677,GO:0003682
6h-Up Reg	*Peaxi162Scf00104g00017*	ERD (early-responsive to dehydration stress) family protein	1.23	9.87	8.00	5.00E-05	1.85E-04	89.09	48.40	3.48E-12	1.44E-10	AT4G02900.1	IPR027815,IPR003864	GO:0016020
6h-Up Reg	*Peaxi162Scf00171g00525*	glutamate decarboxylase	0.35	2.74	7.89	2.29E-02	4.37E-02	19.10	31.81	1.70E-08	3.48E-07	AT5G17330.1	IPR015424,IPR002129	GO:0019752,GO:0016831,GO:0006536,GO:0004351,GO:0003824,GO:0030170
6h-Up Reg	*Peaxi162Scf00102g00178*	Unknown protein	12.73	95.62	7.51	5.00E-05	1.85E-04	3.01	12.15	4.91E-04	2.81E-03			
6h-Up Reg	*Peaxi162Scf00101g00031*	Protein of unknown function, DUF584	2.50	14.97	5.98	3.50E-03	8.56E-03	7.44	25.98	3.45E-07	5.26E-06	AT5G60680.1	IPR007608	
6h-Dn Reg	*Peaxi162Scf00332g10028*	Plant protein of unknown function (DUF247)	3.54	0.33	10.60	8.00E-03	1.76E-02	92.06	53.75	2.27E-13	1.19E-11	AT3G02645.1	IPR004158	
6h-Dn Reg	*Peaxi162Scf00551g00011*	ZCF37, putative [Theobroma cacao]	6.97	0.69	10.17	1.13E-02	2.36E-02	19.27	13.25	2.72E-04	1.70E-03	ref|XP_007029922.1|		
6h-Dn Reg	*Peaxi162Scf00030g00127*	EXORDIUM like 2	4.80	0.49	9.88	3.95E-03	9.52E-03	7.93	15.93	6.58E-05	5.07E-04	AT5G64260.1	IPR006766	
6h-Dn Reg	*Peaxi162Scf00380g00817*	basic helix-loop-helix (bHLH) DNA-binding superfamily protein	4.34	0.56	7.78	9.25E-03	1.99E-02	7.82	9.98	1.58E-03	7.44E-03	AT4G37850.1	IPR011598	GO:0046983
6h-Dn Reg	*Peaxi162Scf00074g00355*	R2R3-MYB transcription factor [Prunus avium]	6.33	0.82	7.71	2.25E-02	4.30E-02	14.13	15.54	8.06E-05	6.02E-04	gb|ADY15314.1|	IPR009057	GO:0003677,GO:0003682
6h-Dn Reg	*Peaxi162Scf01372g00039*	Protein of unknown function (DUF1442)	3.53	0.49	7.24	6.55E-03	1.48E-02	18.97	13.35	2.58E-04	1.63E-03	AT5G62280.1	IPR009902	
6h-Dn Reg	*Peaxi162Scf00980g00018*	Basic helix-loop-helix (bHLH) DNA-binding superfamily protein	2.94	0.67	4.40	2.28E-02	4.35E-02	3.50	15.69	7.45E-05	5.63E-04	AT5G48560.1	IPR011598	GO:0046983
6h-Dn Reg	*Peaxi162Scf00058g00179*	Unknown protein	5.74	1.37	4.19	2.68E-02	5.00E-02	3.70	14.11	1.73E-04	1.15E-03			
6h-Dn Reg	*Peaxi162Scf00258g00114*	Unknown protein	16.26	4.63	3.51	1.80E-03	4.79E-03	2.56	10.35	1.30E-03	6.30E-03			
6h-Dn Reg	*Peaxi162Scf00616g00625*	BTB/POZ domain-containing protein	2.64	0.85	3.10	2.21E-02	4.23E-02	2.77	13.73	2.11E-04	1.37E-03	AT5G60050.1	IPR011333	
	**Gene Short Name**	**Annotation**	**Cufflinks**					**edgeR**				***Arabidopsis* Homolog**	**IPR**	**GO**
			**RT_CTR_48h**	**RT_STR_48h**	**Fold Change**	***p*-Value**	***p*-Value**	**Fold Change**	**LR**	***p*-Value**	**FDR**			
48h-Up Reg	*Peaxi162Scf00672g00810*	GDSL esterase/lipase	3.56	63.04	17.70	5.00E-05	1.85E-04	20.26	63.67	1.47E-15	1.16E-13	sp|Q9FHW9|GDL90_ARATH	IPR001087	GO:0016787,GO:0016788,GO:0006629
48h-Up Reg	*Peaxi162Scf00106g01710*	Cytochrome P450, family 96, subfamily A, polypeptide 10	0.29	4.71	16.00	2.65E-02	4.94E-02	64.53	34.40	4.48E-09	1.21E-07	AT4G39490.1	IPR001128	GO:0020037,GO:0016705,GO:0005506,GO:0055114
48h-Up Reg	*Peaxi162Scf00516g00330*	Palmitoyl-acyl carrier protein thioesterase, chloroplastic	0.37	5.59	15.22	2.65E-02	4.95E-02	17.64	23.08	1.55E-06	2.43E-05	sp|Q9SJE2|FATB_ARATH	IPR002864	GO:0016790,GO:0006633
48h-Up Reg	*Peaxi162Scf00069g01724*	Protein phosphatase 2C family protein	1.42	20.73	14.58	5.00E-05	1.85E-04	18.73	61.31	4.87E-15	3.56E-13	AT3G15260.1	IPR001932,IPR015655	GO:0003824
48h-Up Reg	*Peaxi162Scf00059g01920*	CASP-like protein	10.01	139.24	13.91	5.00E-05	1.85E-04	16.09	59.37	1.30E-14	8.92E-13	sp|A7PJ32|CSPL2_VITVI	IPR006702	
48h-Up Reg	*Peaxi162Scf00037g00185*	Unknown protein	0.91	12.48	13.72	2.50E-04	8.18E-04	18.31	29.13	6.77E-08	1.42E-06			
48h-Up Reg	*Peaxi162Scf00074g01735*	WRKY DNA-binding protein 24	1.39	19.09	13.71	5.85E-03	1.34E-02	13.10	19.77	8.76E-06	1.12E-04	AT5G41570.1	IPR003657	GO:0006355,GO:0043565,GO:0003700
48h-Up Reg	*Peaxi162Scf01058g00011*	Cytochrome P450, family 86, subfamily A, polypeptide 1	5.21	60.55	11.62	5.00E-05	1.85E-04	14.35	25.94	3.52E-07	6.36E-06	AT5G58860.1	IPR001128	GO:0020037,GO:0016705,GO:0005506,GO:0055114
48h-Up Reg	*Peaxi162Scf00083g01919*	Aldehyde dehydrogenase family 3 member F1	0.44	4.92	11.07	1.90E-03	5.02E-03	5.87	6.71	9.61E-03	4.03E-02	sp|Q70E96|AL3F1_ARATH	IPR016161,IPR012394	GO:0016620,GO:0006081,GO:0008152,GO:0055114,GO:0016491,GO:0004030
48h-Up Reg	*Peaxi162Scf00901g00418*	Metal ion binding protein, putative [*Ricinus communis*]	0.66	6.95	10.56	2.64E-02	4.92E-02	112.76	17.10	3.54E-05	3.81E-04	ref|XP_002526528.1|	IPR006121	GO:0046872,GO:0030001
48h-Dn Reg	*Peaxi162Scf00047g02040*	FASCICLIN-like arabinogalactan 2	45.62	0.62	73.43	1.65E-03	4.43E-03	160.00	85.31	2.56E-20	4.49E-18	AT4G12730.1	IPR008700,IPR000782	
48h-Dn Reg	*Peaxi162Scf00559g00019*	2-Oxoglutarate (2OG) and Fe^II^-dependent oxygenase superfamily	61.64	1.00	61.82	5.00E-05	1.85E-04	86.97	75.19	4.27E-18	5.00E-16	AT3G12900.1	IPR002283,IPR027443,IPR026992	GO:0016706,GO:0005506,GO:0055114,GO:0016491
48h-Dn Reg	*Peaxi162Scf72209g00001*	Unknown protein	13.41	0.41	33.04	2.52E-02	4.73E-02	63.68	28.31	1.03E-07	2.09E-06			
48h-Dn Reg	*Peaxi162Scf00195g01222*	Unknown protein	110.22	3.73	29.57	6.50E-03	1.47E-02	237.25	25.02	5.69E-07	9.79E-06			
48h-Dn Reg	*Peaxi162Scf01049g00041*	Unknown protein	5.14	0.23	22.65	1.01E-02	2.14E-02	73.82	32.17	1.41E-08	3.47E-07			
48h-Dn Reg	*Peaxi162Scf00117g00052*	UDP-glycosyltransferase superfamily protein	4.60	0.21	22.09	2.65E-03	6.71E-03	25.50	26.80	2.26E-07	4.21E-06	AT4G15480.1	IPR002213	GO:0008152,GO:0016758
48h-Dn Reg	*Peaxi162Scf00206g00311*	Expansin B3	17.99	0.89	20.20	5.00E-04	1.53E-03	17.81	38.66	5.05E-10	1.63E-08	AT4G28250.1	IPR007118	GO:0019953,GO:0005576
48h-Dn Reg	*Peaxi162Scf00665g00142*	Xyloglucan endotransglucosylase/hydrolase 26	14.12	0.81	17.39	1.05E-03	2.97E-03	25.99	11.02	9.03E-04	6.04E-03	AT4G28850.1	IPR016455,IPR008264,IPR008985	GO:0048046,GO:0016762,GO:0004553,GO:0005618,GO:0006073,GO:0005975
48h-Dn Reg	*Peaxi162Scf00025g00283*	Transducin/WD40 repeat-like superfamily protein	4.57	0.26	17.30	2.65E-03	6.71E-03	25.81	44.36	2.73E-11	1.11E-09	AT5G23730.1	IPR015943	GO:0005515
48h-Dn Reg	*Peaxi162Scf00326g00712*	Peroxidase superfamily protein	25.15	1.52	16.59	5.00E-05	1.85E-04	17.04	18.30	1.89E-05	2.20E-04	AT1G30870.1	IPR010255	GO:0006979,GO:0020037,GO:0004601,GO:0055114

**^1^**LR = likelihood ratio; ^2^FDR = false discovery rate; ^3^IPR = InterPro protein identifier; ^4^GO = gene ontology terms molecular function.

**Table 2:** Top 17 up- and downregulated, and 20 downregulated DEGs.

Annotation	Leaf 6 h				Leaf 24 h				Root 6 h				Root 12 h				Root 24 h				Root 48 h			
	Cufflinks		edgeR		Cufflinks		edgeR		Cufflinks		edgeR		Cufflinks		edgeR		Cufflinks		edgeR		Cufflinks		edgeR	
	FC	*q*-Value	FC	FDR	FC	*q*-Value	FC	FDR	FC	*q*-Value	FC	FDR	FC	*q*-Value	FC	FDR	FC	*q*-Value	FC	FDR	FC	*q*-Value	FC	FDR
BEL1-like homeodomain 6	1.7	3.80E-03	1.6	3.31E-03	1.7	4.27E-03	1.6	7.24E-03	1.5	1.75E-02	1.8	3.07E-04	1.7	2.72E-03	1.8	3.07E-04	1.8	3.54E-04	1.8	2.08E-04	1.7	4.08E-03	2.0	9.05E-05
Cold-regulated 413-plasma membrane 2	5.3	1.75E-04	5.4	2.83E-11	4.0	1.75E-04	3.6	6.01E-07	2.8	1.85E-04	4.2	3.05E-08	3.2	1.85E-04	4.2	3.05E-08	1.8	1.25E-03	2.4	4.87E-04	2.7	1.85E-04	3.2	2.29E-05
Basic helix-loop-helix (bHLH) DNA-binding	2.0	1.75E-04	1.8	1.86E-03	1.6	4.50E-03	1.6	1.02E-02	3.2	1.85E-04	1.8	1.21E-03	1.6	1.10E-02	1.8	1.21E-03	3.1	1.85E-04	1.8	7.63E-04	2.2	1.85E-04	2.8	8.79E-07
Protein NRT1/PTR FAMILY 1.2	2.2	1.75E-04	2.2	2.22E-03	3.0	1.75E-04	3.3	3.24E-06	1.7	2.12E-02	2.6	4.27E-04	1.9	6.71E-03	2.6	4.27E-04	3.1	1.25E-03	4.7	4.12E-08	1.6	4.45E-02	2.0	2.30E-02
Pleckstrin (PH) domain-containing protein	3.9	1.75E-04	4.3	1.24E-12	30.9	1.75E-04	40.1	1.12E-56	2.4	1.85E-04	2.5	2.37E-05	1.7	1.94E-03	2.5	2.37E-05	3.1	1.85E-04	3.2	1.50E-08	2.3	1.85E-04	2.8	2.39E-06
Unknown protein	2.2	1.75E-04	2.1	4.16E-05	4.9	1.75E-04	4.9	1.16E-19	2.4	3.54E-04	2.3	5.40E-06	2.0	6.68E-04	2.3	5.40E-06	1.6	9.20E-03	2.0	4.75E-05	2.4	1.85E-04	2.5	2.85E-07
NAD(P)-binding Rossmann-fold	7.4	1.75E-04	7.0	5.98E-16	6.6	1.75E-04	7.0	1.44E-15	2.1	1.85E-04	3.1	5.66E-06	2.7	1.85E-04	3.1	5.66E-06	5.5	1.85E-04	5.7	7.46E-13	3.6	1.85E-04	3.7	3.95E-07
Plant invertase/pectin methylesterase	3.2	1.75E-04	2.9	3.51E-07	2.3	1.75E-04	2.6	6.70E-06	2.1	1.85E-04	1.7	1.41E-02	2.6	1.85E-04	1.7	1.41E-02	2.3	1.85E-04	1.8	7.46E-03	2.5	1.85E-04	3.3	1.11E-06
Dehydration-responsive element-binding	4.6	1.75E-04	4.7	1.89E-04	5.5	1.75E-04	6.1	1.24E-05	3.7	1.85E-04	9.6	8.42E-07	6.9	3.54E-04	9.6	8.42E-07	4.7	1.80E-03	8.5	1.73E-06	5.8	3.47E-03	7.4	1.90E-04
Sec14p-like phosphatidylinositol transfer	2.1	1.75E-04	2.0	1.79E-02	4.5	1.75E-04	4.6	1.46E-08	3.0	1.85E-04	2.3	1.16E-02	2.3	3.54E-04	2.3	1.16E-02	2.8	3.54E-04	4.1	9.30E-06	1.7	1.98E-02	2.3	2.03E-02
Major facilitator superfamily protein	2.0	1.75E-04	2.2	5.73E-04	2.4	1.75E-04	2.5	4.34E-05	2.3	1.85E-04	7.0	1.15E-16	4.7	1.85E-04	7.0	1.15E-16	3.9	1.85E-04	4.4	3.14E-10	6.3	1.85E-04	8.9	3.99E-15
Trehalose phosphate synthase	2.4	1.75E-04	2.5	1.62E-03	2.9	1.75E-04	2.6	9.50E-04	1.7	3.34E-03	3.4	1.70E-05	2.7	1.85E-04	3.4	1.70E-05	5.5	1.85E-04	5.4	9.51E-10	2.3	1.85E-04	2.7	7.49E-04
Glycosyl hydrolase family protein	3.4	1.75E-04	3.6	1.10E-07	4.1	1.75E-04	4.3	8.05E-10	4.1	1.85E-04	2.0	8.91E-03	2.7	1.85E-04	2.0	8.91E-03	2.4	1.85E-04	3.2	2.84E-06	2.4	1.85E-04	2.3	1.67E-03
Heat stress transcription factor C-1	3.4	1.75E-04	3.4	3.91E-08	3.1	1.75E-04	3.0	1.54E-06	8.1	1.85E-04	3.6	2.75E-08	3.8	1.85E-04	3.6	2.75E-08	2.5	1.85E-04	2.7	1.67E-05	3.0	1.85E-04	3.2	5.09E-05
Conserved hypothetical protein	1.5	2.02E-02	1.5	4.44E-02	2.6	1.75E-04	2.4	8.21E-08	1.6	9.73E-03	3.2	3.42E-11	2.4	1.85E-04	3.2	3.42E-11	3.3	1.85E-04	3.5	2.86E-13	2.1	1.85E-04	2.7	1.75E-07
Peroxidase superfamily protein	2.4	1.75E-04	2.3	1.17E-02	2.2	1.75E-04	2.1	2.00E-02	2.5	1.85E-04	4.6	1.38E-06	4.2	1.85E-04	4.6	1.38E-06	6.4	1.85E-04	6.1	5.53E-09	5.7	1.85E-04	5.5	8.27E-08
α/β-Hydrolases	1.8	6.34E-04	1.8	6.61E-03	4.3	1.75E-04	4.3	1.17E-13	2.2	1.85E-04	2.4	5.69E-05	2.1	1.85E-04	2.4	5.69E-05	3.6	1.85E-04	3.9	1.38E-11	2.0	1.85E-04	2.1	7.83E-04
Nucleobase-ascorbate transporter 12	0.6	4.61E-03	0.6	2.37E-02	0.7	1.91E-02	0.6	1.99E-02	0.5	6.18E-03	0.4	7.86E-05	0.4	1.85E-04	0.4	7.86E-05	0.3	1.85E-04	0.3	2.91E-08	0.4	1.85E-04	0.5	2.01E-03
Glycine-rich RNA-binding protein	0.3	1.75E-04	0.4	9.99E-05	0.6	2.61E-03	0.5	6.83E-03	0.2	1.85E-04	0.3	2.24E-09	0.2	1.85E-04	0.3	2.24E-09	0.3	1.85E-04	0.3	1.50E-06	0.4	1.85E-04	0.4	2.59E-05
ACT domain repeat 4	0.6	2.11E-03	0.5	2.64E-03	0.1	1.75E-04	0.0	7.17E-47	0.3	1.85E-04	0.4	2.25E-05	0.4	1.85E-04	0.4	2.25E-05	0.6	1.53E-03	0.7	3.83E-02	0.2	1.85E-04	0.2	7.95E-18
Homeodomain-like superfamily protein	0.4	1.52E-02	0.3	5.53E-04	0.2	3.34E-04	0.3	2.22E-05	0.4	2.03E-02	0.2	1.21E-05	0.4	9.83E-03	0.2	1.21E-05	0.1	4.90E-03	0.2	1.87E-06	0.4	3.56E-02	0.4	2.24E-02
Glucose-6-phosphate dehydrogenase 2	0.6	3.69E-03	0.6	8.22E-03	0.5	1.75E-04	0.5	1.82E-04	0.4	1.85E-04	0.4	5.31E-08	0.3	1.85E-04	0.4	5.31E-08	0.3	1.85E-04	0.4	1.94E-07	0.4	1.85E-04	0.4	1.74E-06
ACT domain repeat 4	0.4	1.75E-04	0.4	1.03E-04	0.4	1.75E-04	0.4	8.36E-04	0.5	6.51E-03	0.3	6.15E-05	0.3	6.68E-04	0.3	6.15E-05	0.4	1.85E-04	0.4	2.38E-03	0.2	1.85E-04	0.2	5.76E-08
Nodulin MtN21/EamA-like transporter	0.5	1.22E-02	0.5	6.71E-03	0.2	1.75E-04	0.2	4.98E-13	0.5	4.43E-03	0.4	7.14E-05	0.3	1.85E-04	0.4	7.14E-05	0.3	1.85E-04	0.4	1.42E-05	0.4	1.85E-04	0.4	1.51E-04
Protein COBRA	0.3	1.75E-04	0.3	9.82E-08	0.2	1.75E-04	0.2	2.49E-14	0.3	8.77E-03	0.3	1.16E-04	0.3	2.84E-03	0.3	1.16E-04	0.3	1.85E-04	0.3	1.21E-05	0.4	1.53E-03	0.4	2.30E-03
Ferredoxin-3, chloroplastic	0.3	4.86E-04	0.3	3.74E-06	0.3	1.98E-03	0.4	1.88E-04	0.5	1.85E-04	0.3	1.15E-07	0.3	1.85E-04	0.3	1.15E-07	0.1	1.85E-04	0.2	3.99E-14	0.1	1.85E-04	0.1	2.68E-18
Fatty acid desaturase 2	0.6	3.34E-04	0.6	1.44E-02	0.3	1.75E-04	0.3	1.32E-07	0.5	1.67E-03	0.5	1.15E-03	0.4	1.85E-04	0.5	1.15E-03	0.2	1.85E-04	0.3	2.39E-10	0.3	1.85E-04	0.3	7.93E-10
Ferredoxin—NADP reductase, root isozyme	0.1	1.75E-04	0.1	1.15E-33	0.5	1.75E-04	0.5	2.43E-03	0.5	1.85E-04	0.4	3.52E-06	0.4	1.85E-04	0.4	3.52E-06	0.3	1.85E-04	0.3	4.63E-10	0.4	1.85E-04	0.5	2.27E-03
Xyloglucan endotransglucosylase/hydrolase	0.1	1.75E-04	0.1	1.18E-07	0.2	1.75E-04	0.2	6.29E-05	0.1	1.85E-04	0.1	1.39E-14	0.0	1.85E-04	0.1	1.39E-14	0.0	1.85E-04	0.0	2.27E-17	0.1	1.85E-04	0.1	7.61E-11
Fatty acid hydroxylase superfamily	0.3	3.34E-04	0.2	2.22E-09	0.3	3.34E-04	0.3	1.58E-08	0.5	1.11E-02	0.3	1.03E-05	0.3	3.54E-04	0.3	1.03E-05	0.1	1.01E-02	0.1	1.23E-16	0.2	2.39E-02	0.2	1.33E-10
3-Oxo-5-α-steroid 4-dehydrogenase	0.3	1.75E-04	0.2	3.37E-09	0.3	1.75E-04	0.3	3.63E-08	0.2	2.29E-02	0.1	3.96E-10	0.1	2.72E-03	0.1	3.96E-10	0.2	1.85E-04	0.1	1.20E-13	0.2	1.85E-04	0.2	3.52E-08
Unknown protein	0.5	3.34E-04	0.5	4.33E-03	0.3	1.75E-04	0.3	1.16E-10	0.5	6.68E-04	0.2	2.42E-12	0.2	1.85E-04	0.2	2.42E-12	0.2	1.85E-04	0.3	1.31E-08	0.1	1.85E-04	0.1	1.03E-22
Phosphoenolpyruvate carboxylase 1	0.2	1.75E-04	0.2	2.31E-11	0.4	1.75E-04	0.4	6.10E-05	0.4	1.85E-04	0.3	5.72E-08	0.3	1.85E-04	0.3	5.72E-08	0.3	1.85E-04	0.3	2.16E-09	0.2	1.85E-04	0.2	2.81E-16
Protein phosphatase 2C family protein	0.3	1.75E-04	0.3	2.83E-09	0.3	1.75E-04	0.2	1.36E-12	0.2	1.85E-04	0.2	2.03E-10	0.2	1.85E-04	0.2	2.03E-10	0.2	1.85E-04	0.2	8.05E-15	0.3	1.85E-04	0.4	9.01E-05
Protein NRT1/PTR FAMILY 6.1	0.4	1.75E-04	0.4	3.99E-04	0.3	1.75E-04	0.3	5.75E-05	0.2	1.94E-03	0.2	1.28E-06	0.2	4.20E-03	0.2	1.28E-06	0.1	2.46E-03	0.1	3.80E-10	0.2	4.20E-03	0.2	8.55E-07
Transmembrane amino acid transporter	0.5	6.34E-04	0.5	5.23E-03	0.6	1.63E-02	0.6	3.48E-02	0.6	2.67E-02	0.4	1.00E-04	0.3	1.85E-04	0.4	1.00E-04	0.1	1.85E-04	0.1	1.16E-18	0.3	1.85E-04	0.3	9.29E-06
Tyrosine phosphatase family protein	0.4	2.33E-02	0.2	2.37E-06	0.2	1.19E-03	0.2	1.27E-08	0.2	1.85E-04	0.5	1.22E-02	0.3	1.85E-04	0.5	1.22E-02	0.3	1.85E-04	0.5	6.36E-03	0.3	1.85E-04	0.4	1.29E-04

## Supplementary Materials

The following are available online at www.mdpi.com/2073-4425/8/8/195/s1. Figure S1: Venn diagrams of the DEGs identified with Cufflinks (blue) and edgeR (orange), Intersection of DEGs (used for downstream analysis) is red-colored with white number; Figure S2: The transcriptomic profiles of the samples exposed to NaCl are more similar, (A) Dendrogram based on hierarchical clustering by Pearson using Reads Per Kilobase of transcript per Million mapped reads (RPKM) expression values from all detected genes in all samples. (B) Dendrogram using Spearman rank correlation using RNA-seq (RPKM) expression values from all detected genes in all samples; Figure S3: Two-way Venn diagrams from differentially expressed genes (DEGs) using Cufflinks and edgeR (FDR < 0.05). (A) Venn diagram showing DEGs identified between the LF_CTR_00h/LF_STR_00h (LF_DEGs_00h) and RT_CTR_00h/RT_STR_00h (RT_DEGs_00h). (B) Intersection of the 828 roots DEGs at all time points the 2170 leaf (LF) DEGs (A). (C) Intersection of A and B; Figure S4: Phylogenic tree (including non-plant organisms) showing the *Petunia Hybrida* cv. ‘Mitchell Diploid’ *TPS1* relative to *TPS1* protein sequences from other species: *Nicotiana tabacum*, *Solanum lycopersicum, Vitis vinifera, Populus trichocarpa, Arabidopsis thaliana, Ricinus communis, Sorghum bicolor, Zea mays, Oryza sativa, Triticum aestivum, Selaginella lepidophylla, Candida albicans, Saccharomyces cerevisiae, Clavispora lusitaniae, Apis mellifera, Drosophila melanogaster, Escherichia coli* and *Ginkgo biloba*; Table S1: Summary of RNA-seq data. LF = Leaf, RT = Roots. CTR = No NaCl, STR = 150 mM NaCl. 00h = 0 h of treatment or control, 06h = 6 h of treatment or control, 12h = 12 h of treatment or control, 24h = 24 h of treatment or control and 48h = 48 h of treatment or control. Three biological replicates are indicated as B1, B2 and B3; Table S2: All expressed protein-coding and non protein-coding genes identified at 0 h (00h) of 150 mM salt stress (STR) and control (CTR) treatments in Petunia leaf. Gene ID, annotation and Fragments Per Kilobase of transcript per Million mapped reads (FPKM) are included; Table S3: All expressed protein-coding and non-protein-coding genes identified at 6 h (06h) of 150 mM salt stress (STR) and control (CTR) treatments in petunia leaf, Gene ID, annotation and Fragments Per Kilobase of transcript per Million mapped reads (FPKM) are included; Table S4: All expressed protein-coding and non-protein-coding genes identified at 24 h (24 h) of 150 mM salt stress (STR) and control (CTR) treatments in petunia leaf, Table S5: All expressed protein-coding and non- protein-coding genes identified at 0 h (00h) of 150 mM salt stress (STR) and control (CTR) treatments in Petunia roots. Gene ID, annotation and Fragments Per Kilobase of transcript per Million mapped reads (FPKM) are included; Table S6: All expressed protein-coding and non-protein-coding genes identified at 6 h (06h) of 150 mM salt stress (STR) and control (CTR) treatments in petunia roots. Gene ID, annotation and Fragments Per Kilobase of transcript per Million mapped reads (FPKM) are included; Table S7: All expressed protein-coding and non-protein-coding genes identified at 12 h (12 h) of 150 mM salt stress (STR) and control (CTR) treatments in petunia roots, Gene ID, annotation and Fragments Per Kilobase of transcript per Million mapped reads (FPKM) are included; Table S8: All expressed protein-coding and non-protein-coding genes identified at 24 h (24 h) of 150 mM salt stress (STR) and control (CTR) treatments in petunia roots. Gene ID, annotation and Fragments Per Kilobase of transcript per Million mapped reads (FPKM) are included; Table S9. All expressed protein-coding and non-protein-coding genes identified at 48 h (48 h) of 150 mM salt stress (STR) and control (CTR) treatments in petunia roots, Gene ID, annotation and Fragments Per Kilobase of transcript per Million mapped reads (FPKM) are included; Table S10: Differentially expressed genes (DEGs) commonly identified by Cufflinks and edgeR between Control (CTR) and Salt (STR) in leaf at 0 h (00h) of treatment. A cutoff of 0.05 was used. LF = leaf, RT = roots; Table S11: Differentially expressed genes (DEGs) commonly identified by Cufflinks and edgeR between control (CTR) and salt (STR) in leaf at 6 h (06h) of treatment. A cutoff of 0.05 was used. LF = leaf, RT = roots; Table S12: Differentially expressed genes (DEGs) commonly identified by Cufflinks and edgeR between Control (CTR) and Salt (STR) in leaf at 24h (24 h) of treatment. A cutoff of 0.05 was used. LF = leaf, RT = roots; Table S13: Differentially expressed genes (DEGs) commonly identified by Cufflinks and edgeR between control (CTR) and salt (STR) in root at 0 h (00h) of treatment. A cutoff of 0.05 was used. LF = leaf, RT = roots; Table S14: Differentially expressed genes (DEGs) commonly identified by Cufflinks and edgeR between control (CTR) and salt (STR) in root at 6 h (06h) of treatment. A cutoff of 0.05 was used. LF = leaf, RT = roots; Table S15: Differentially expressed genes (DEGs) commonly identified by Cufflinks and edgeR between control (CTR) and salt (STR) in root at 12 h (12 h) of treatment. A cutoff of 0.05 was used. LF = leaf, RT = roots; Table S16: Differentially expressed genes (DEGs) commonly identified by Cufflinks and edgeR between control (CTR) and salt (STR) in root at 24 h (24 h) of treatment. A cutoff of 0.05 was used. LF = leaf, RT = roots; Table S17: Differentially expressed genes (DEGs) commonly identified by Cufflinks and edgeR between control (CTR) and salt (STR) in root at 48h (48 h) of treatment. A cutoff of 0.05 was used. LF = leaf, RT = roots; Table S18: All 597 identified DEGs that are expressed as an early (06h) response to salt stress (150 mM) in roots, Table S19: All 788 identified DEGs that are expressed as an early (48 h) response to salt stress (150 mM) in roots, Table S20: Differentially expressed genes (control DEGs) from roots (RT) and leaf (LF) at 0 h (00h) of salt stress, Table S21: All the 160 “cleaned” differentially expressed genes DEGs.
