# Supplementary figures and images for: Dissecting Tissue-Specific Transcriptomic Responses from Leaf and Roots under Salt Stress in Petunia hybrida Mitchell
Source: Genes (Basel). 2017 Aug 3;8(8):195. doi: 10.3390/genes8080195 (PMC5575659; doi:10.3390/genes8080195)

LF\_00h

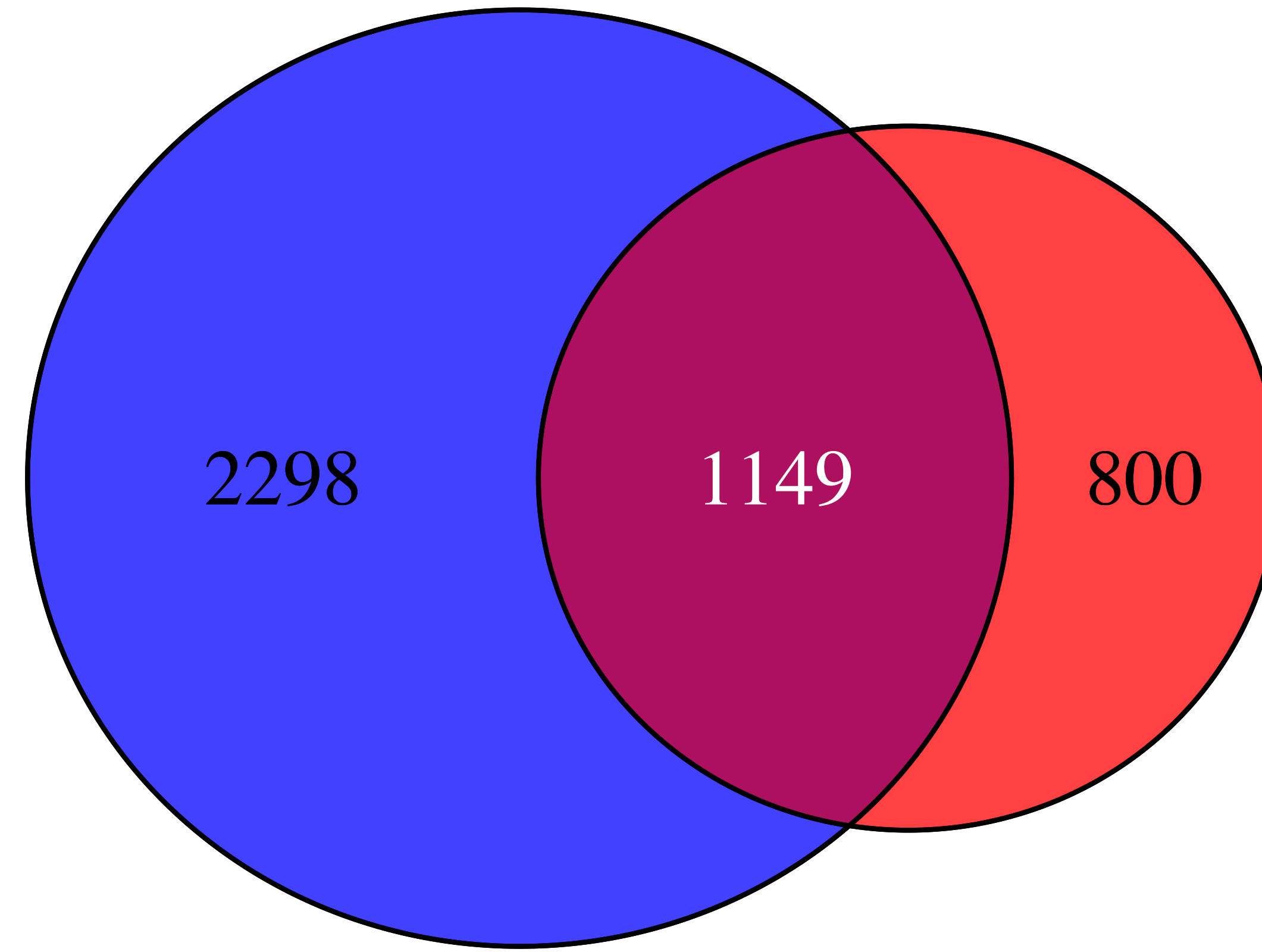

LF\_06h

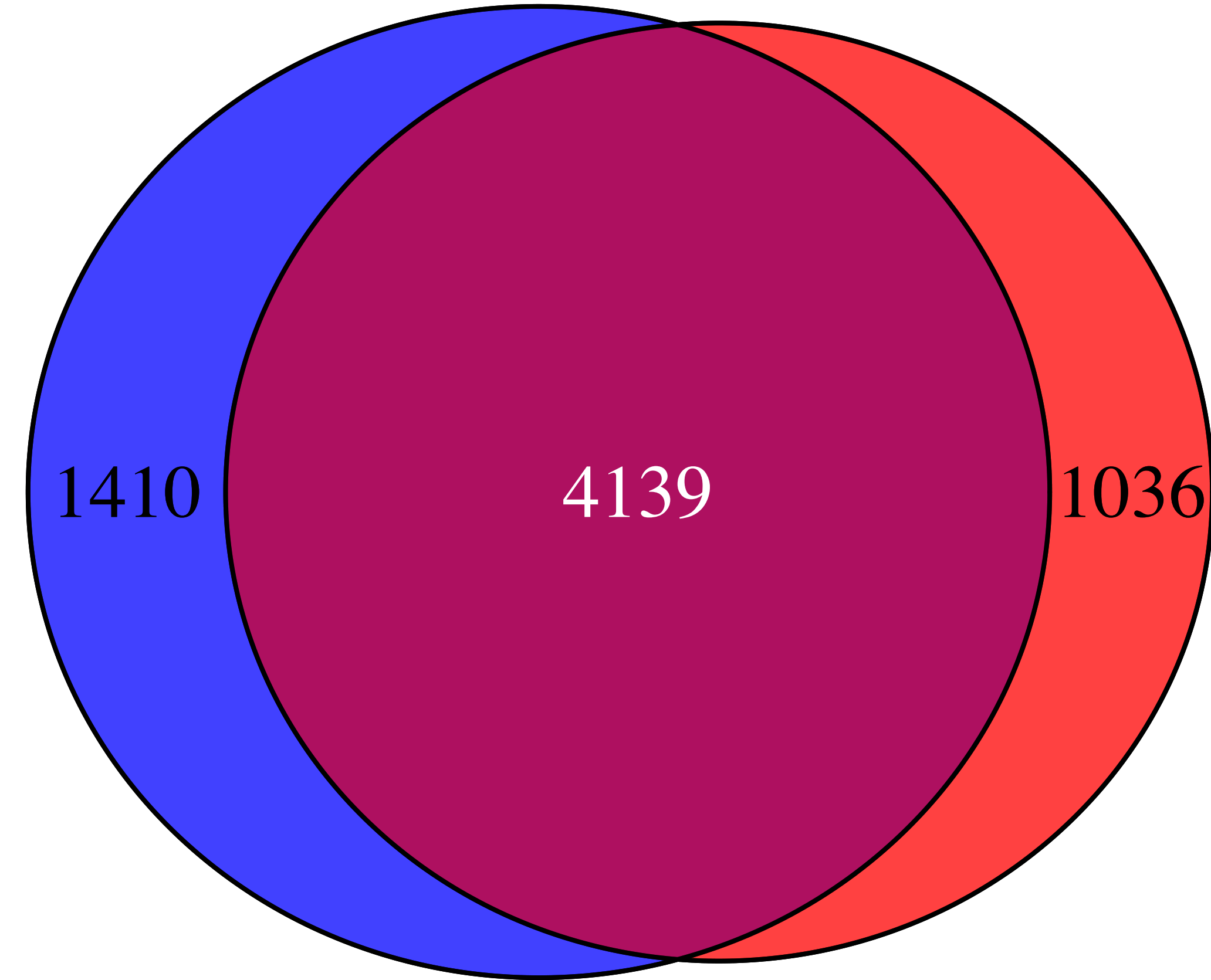

LF\_24h

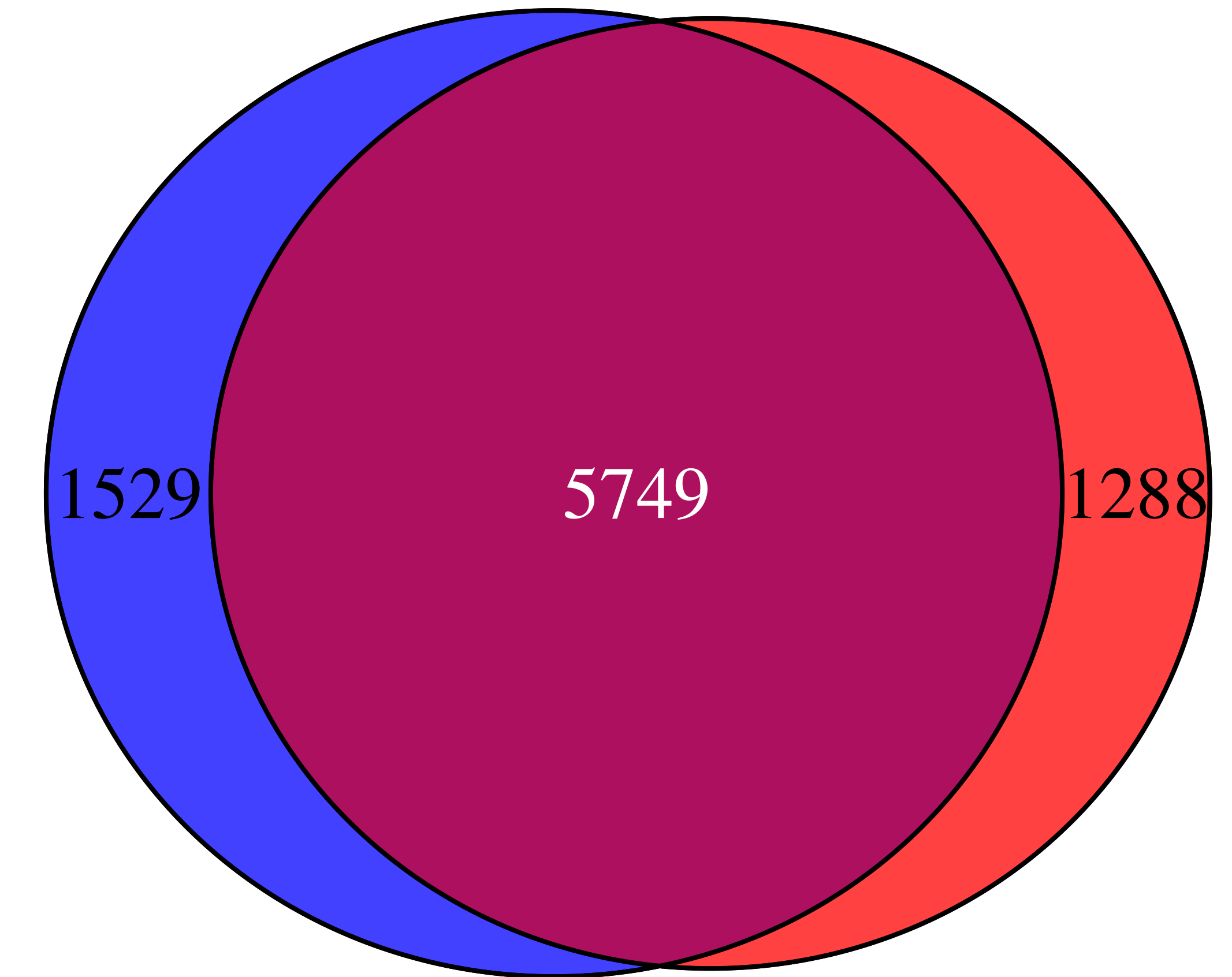

RT\_00h

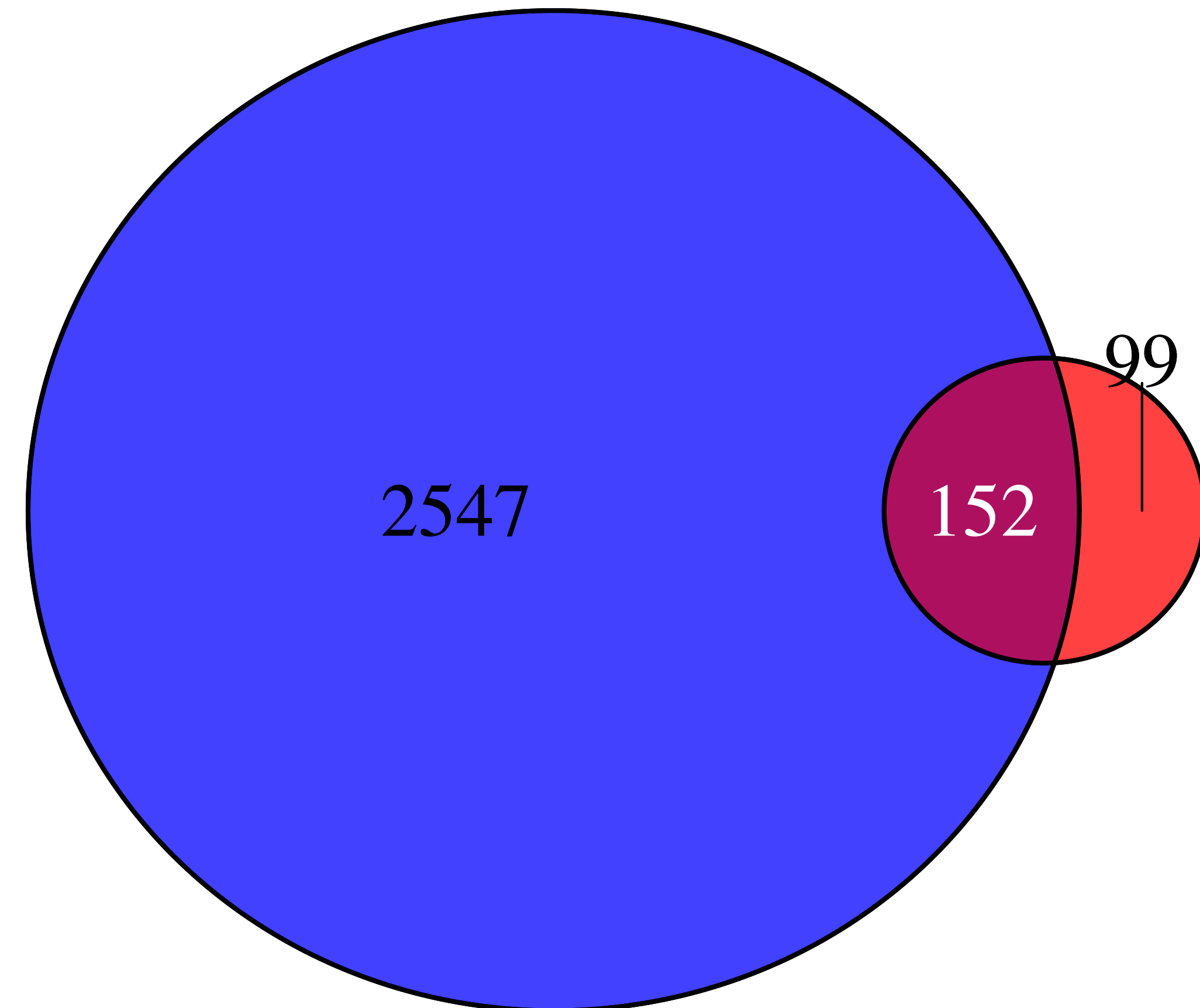

RT\_06h

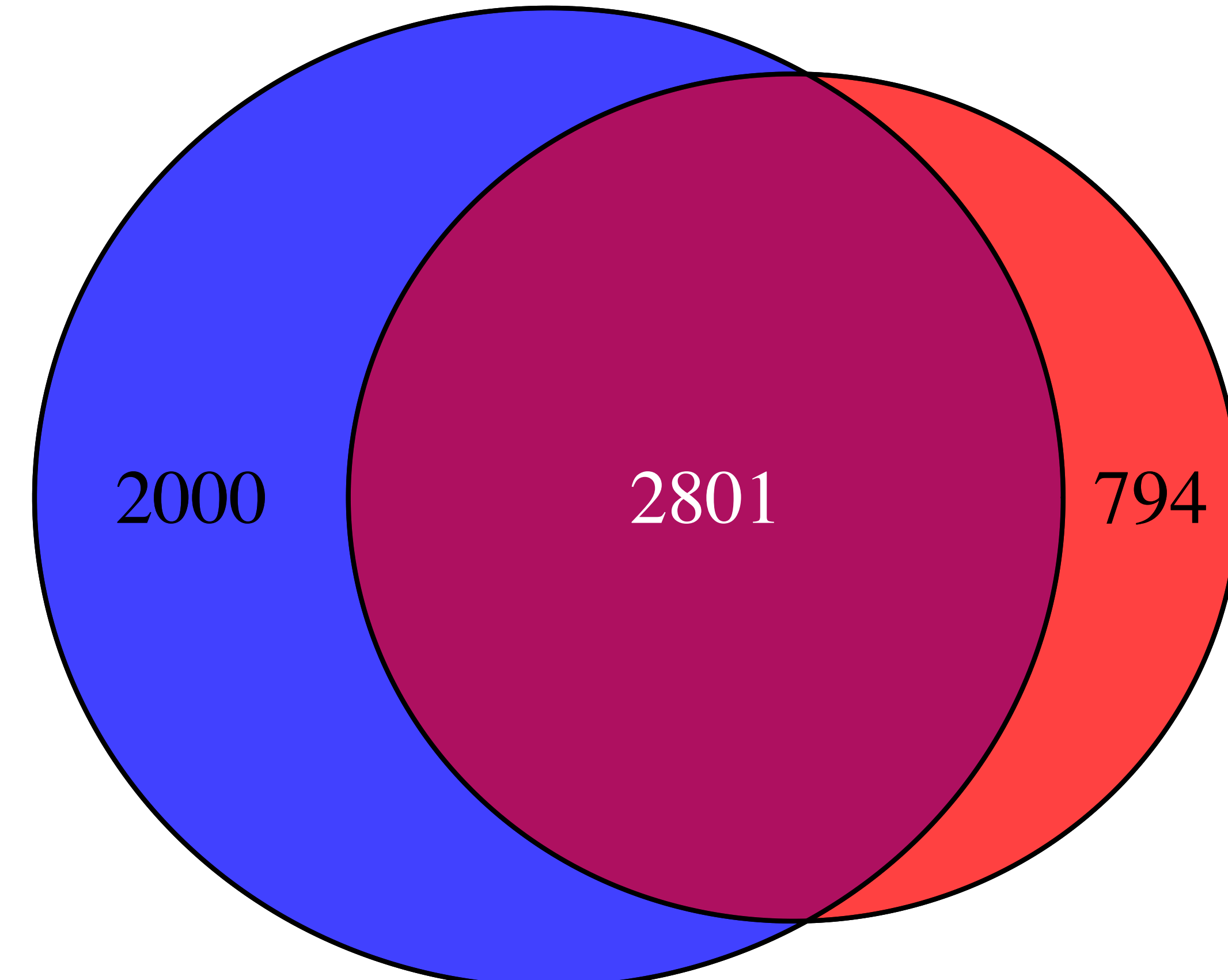

RT\_12h

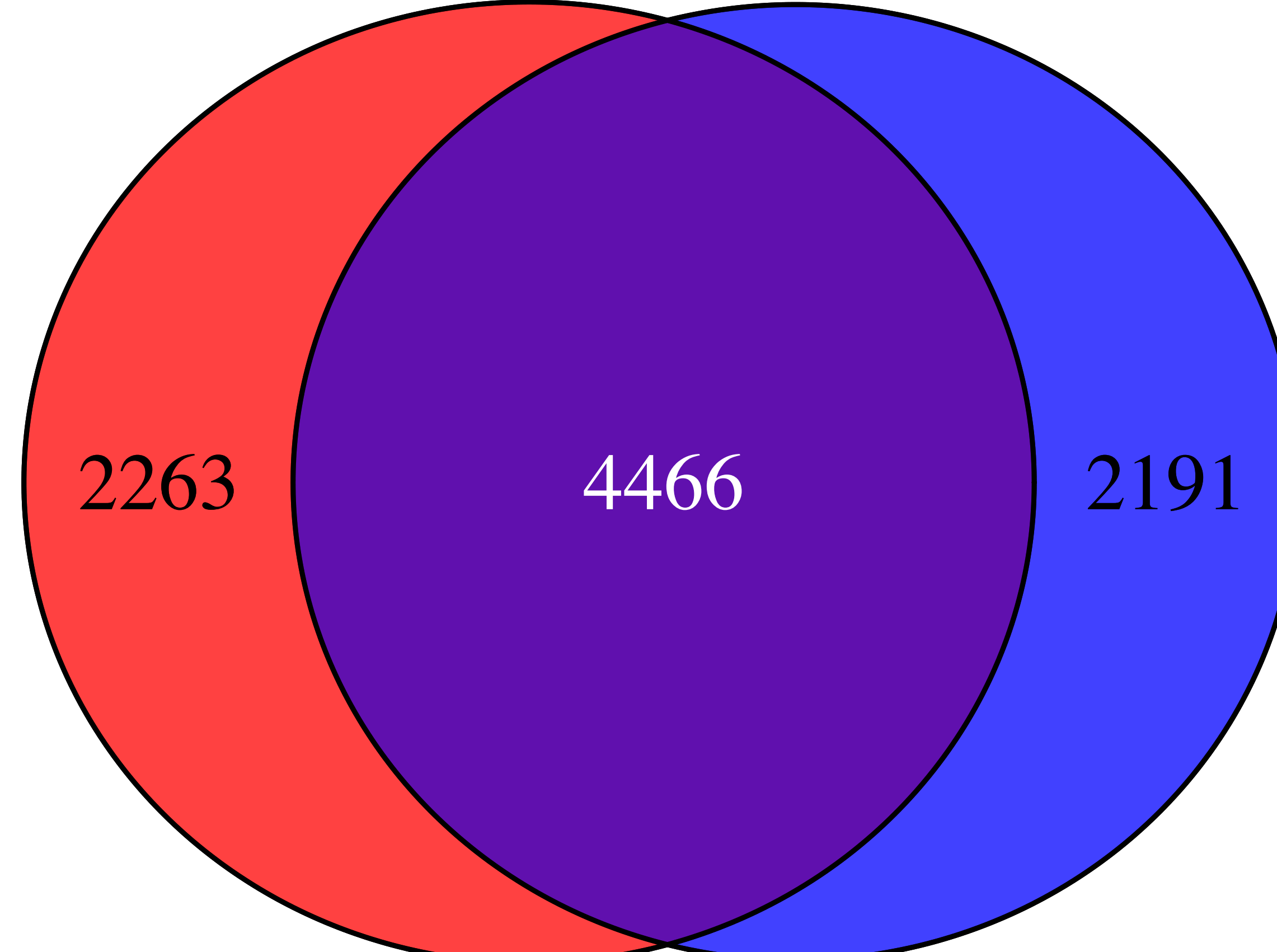

RT\_24h

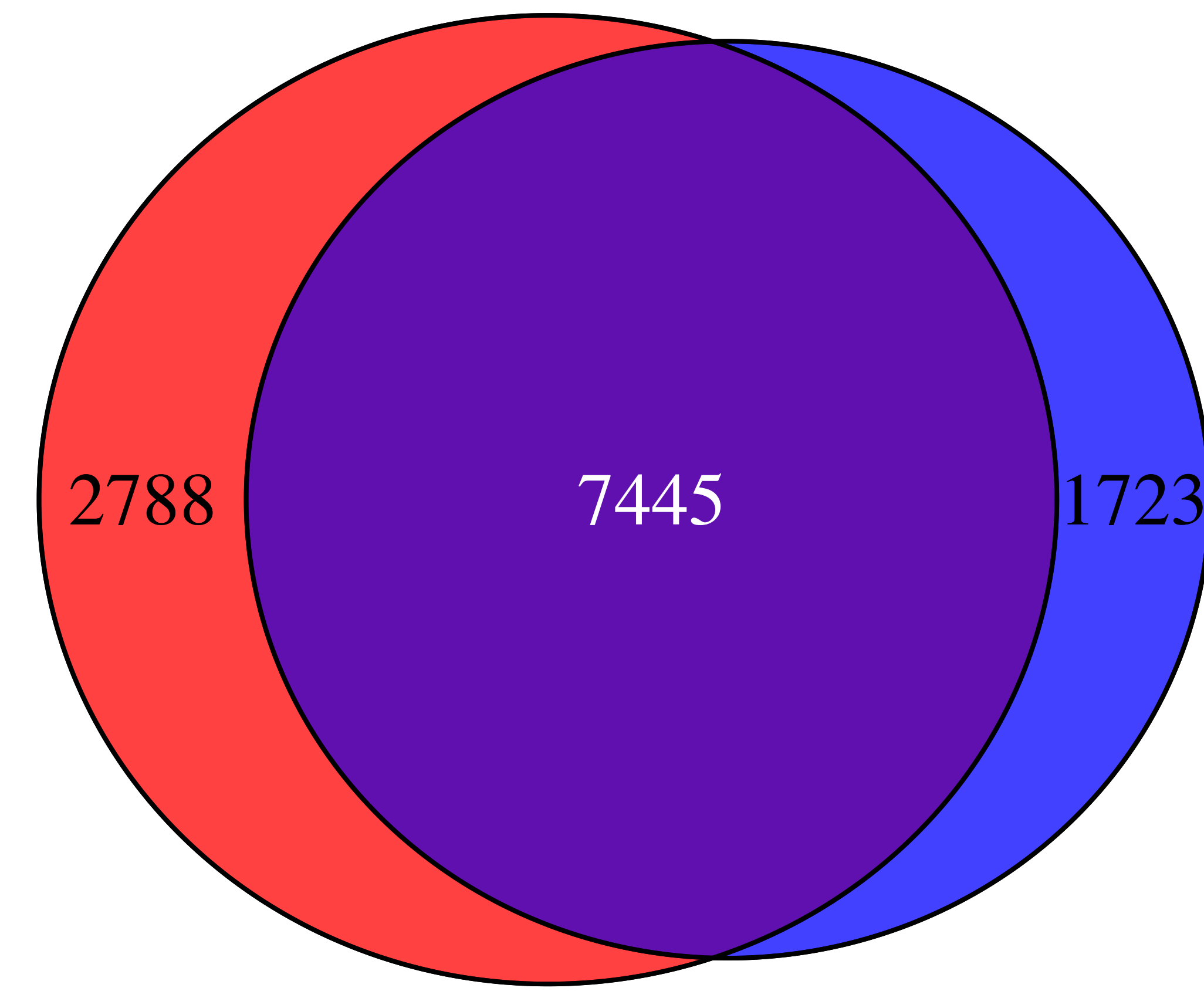

RT\_48h

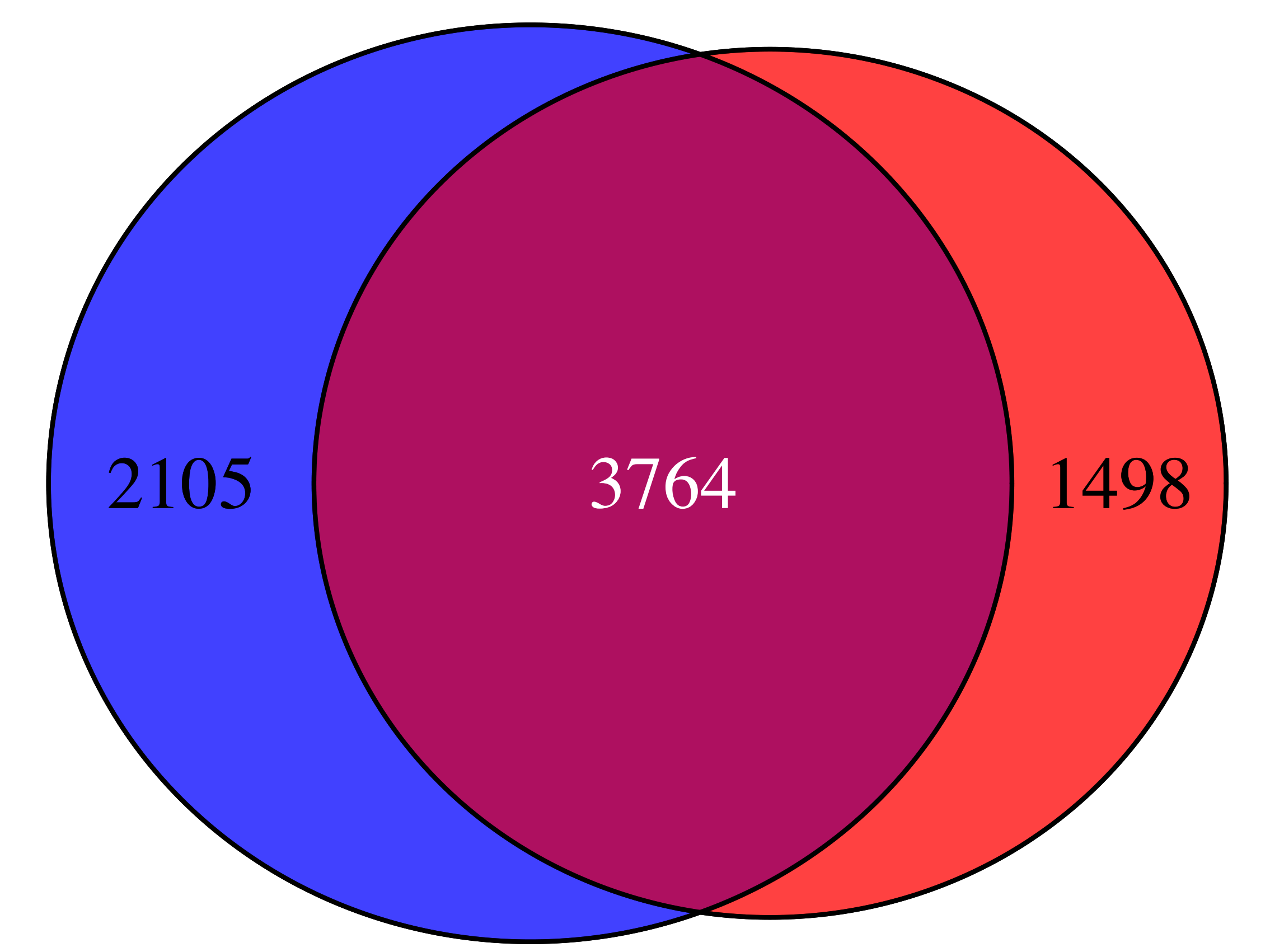

Supplement: Supplementary file 1 [file genes-08-00195-s001.zip › Supplementary Figures and Tables/SupplementaryFigs/Supplemental Fig.1.pdf]

A

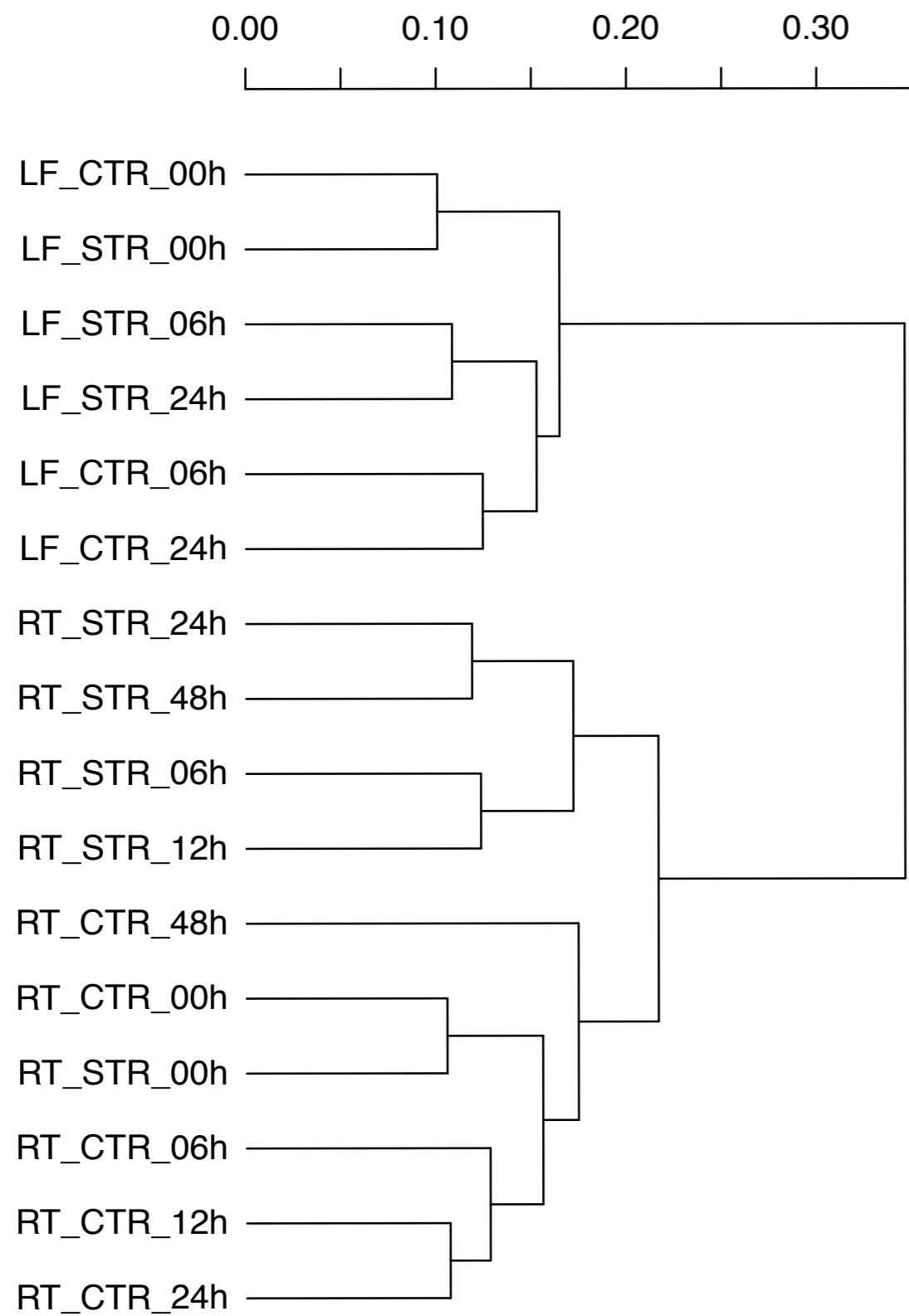

B

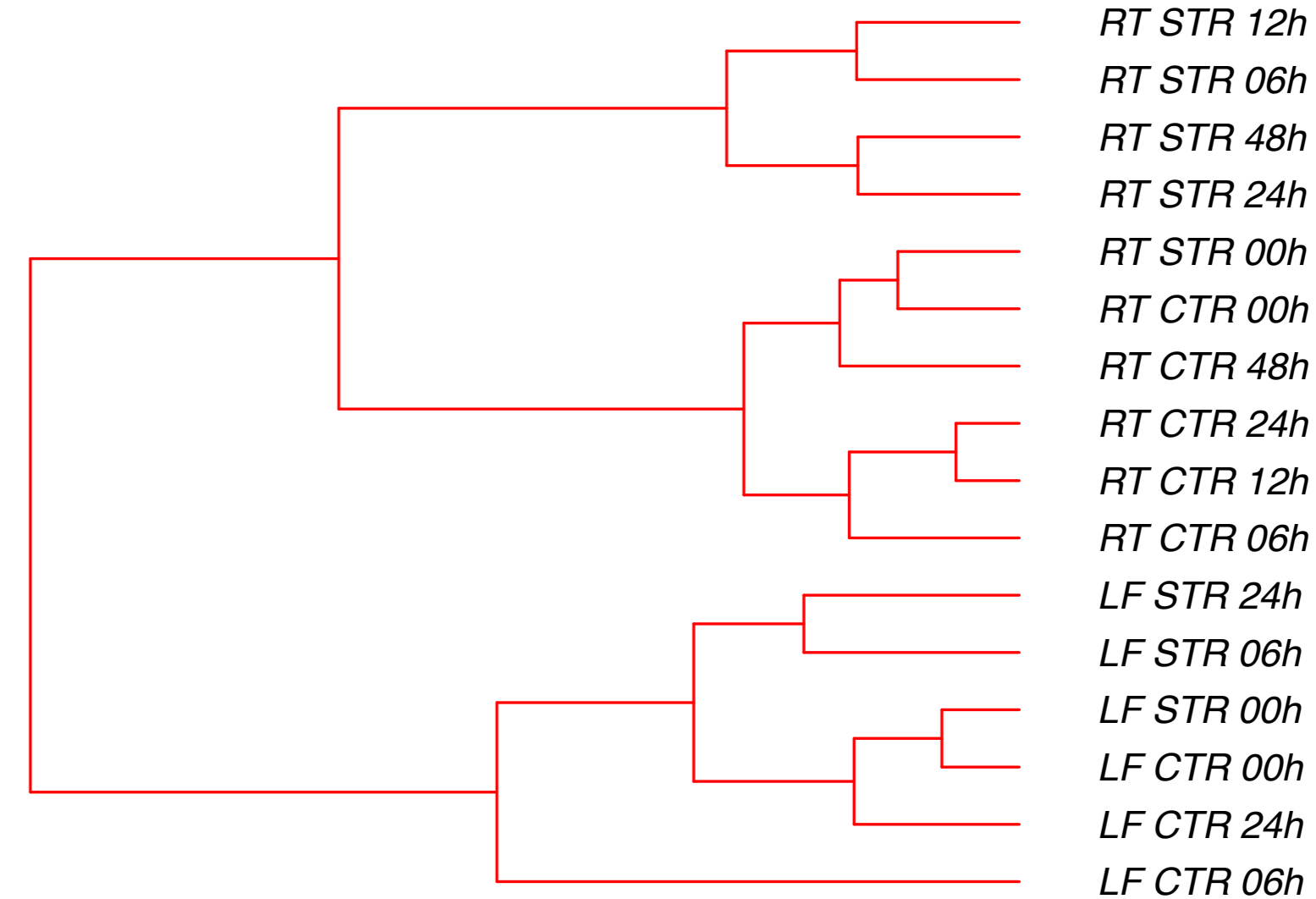

Supplement: Supplementary file 1 [file genes-08-00195-s001.zip › Supplementary Figures and Tables/SupplementaryFigs/Supplemental Fig.2.pdf]

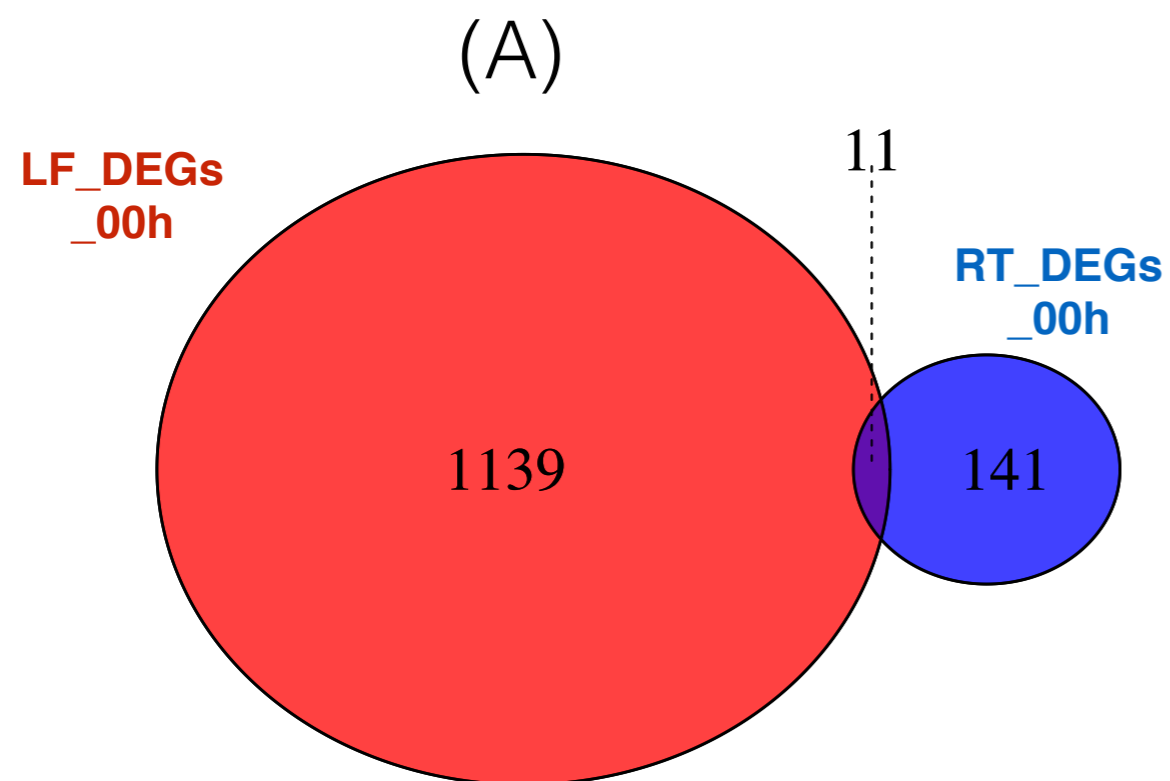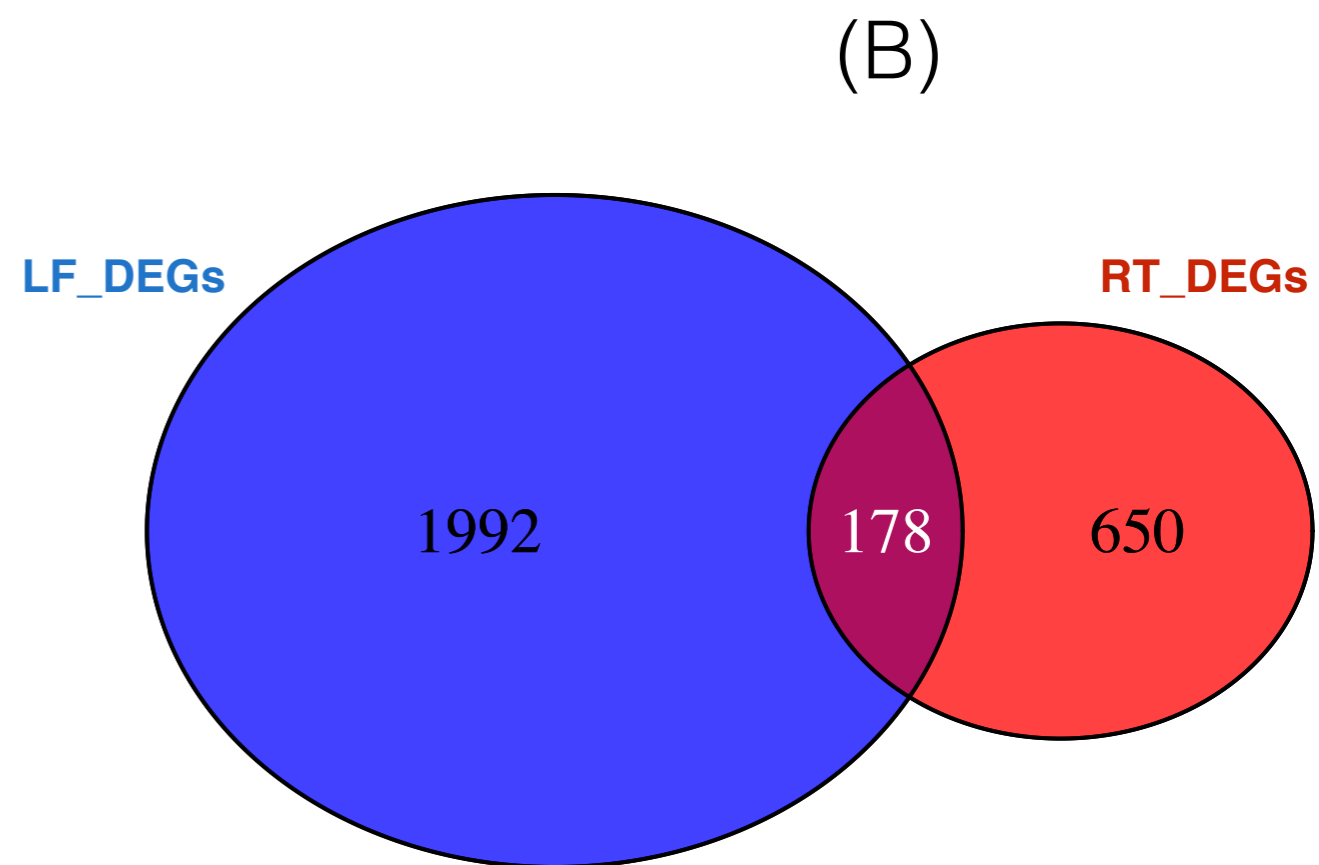

“Union”

“Intersection”

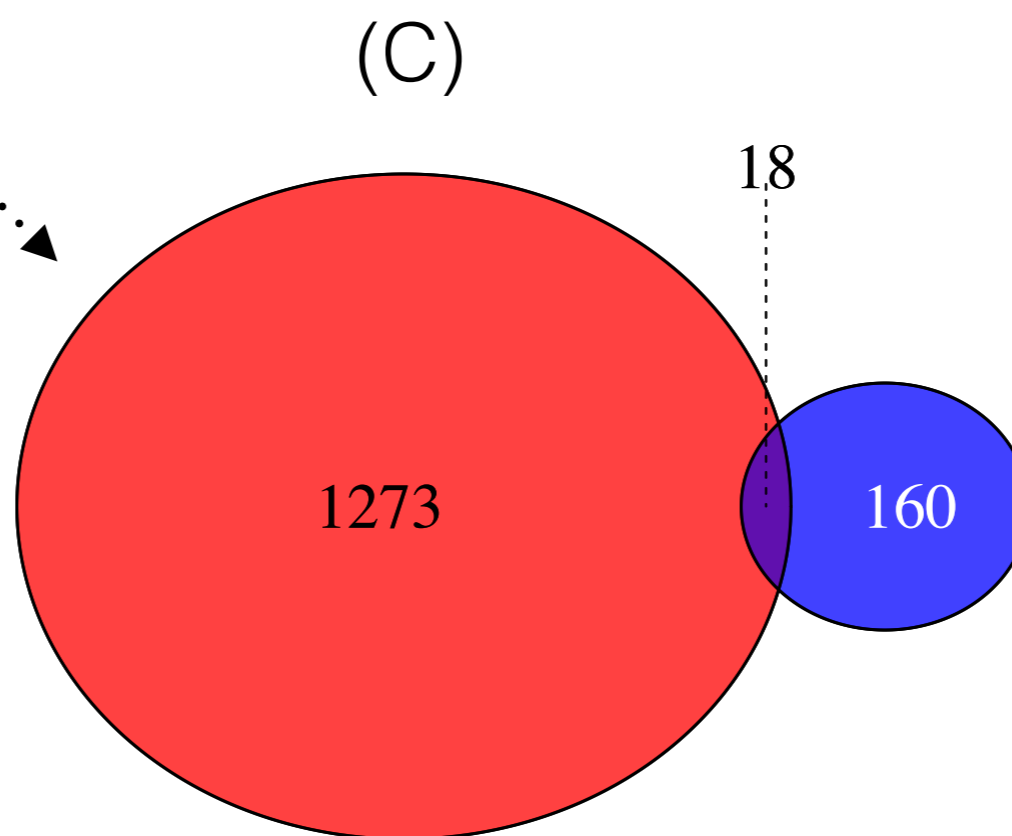

Supplement: Supplementary file 1 [file genes-08-00195-s001.zip › Supplementary Figures and Tables/SupplementaryFigs/Supplemental Fig.3.pdf]

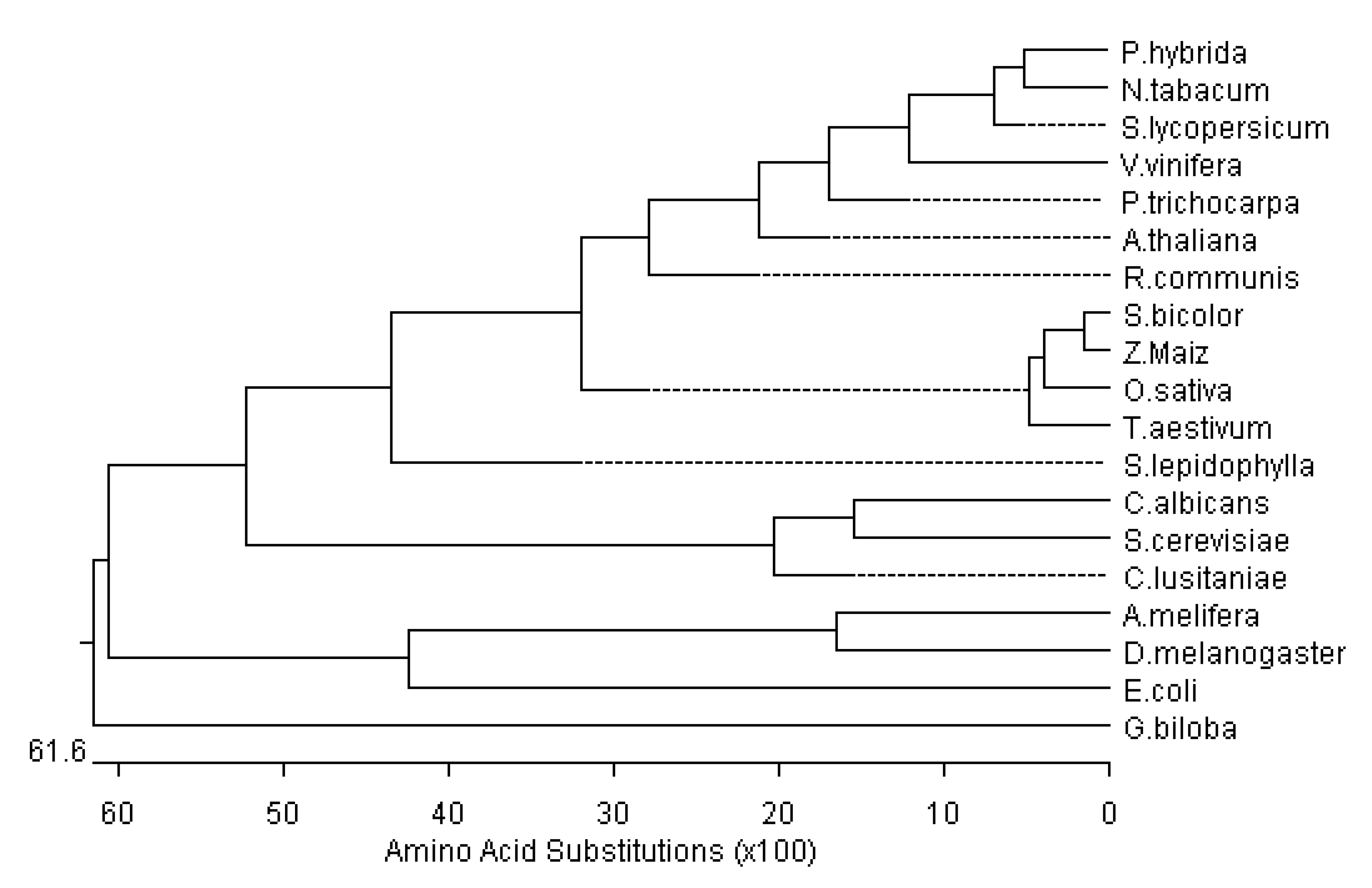

Supplement: Supplementary file 1 [file genes-08-00195-s001.zip › Supplementary Figures and Tables/SupplementaryFigs/Supplemental Fig.4.png]
